# Effect of High Hydrostatic Pressure on the Extractability and Bioaccessibility of Carotenoids and Their Esters from Papaya (*Carica papaya* L.) and Its Impact on Tissue Microstructure

**DOI:** 10.3390/foods10102435

**Published:** 2021-10-13

**Authors:** Sara Lara-Abia, Jorge Welti-Chanes, M. Pilar Cano

**Affiliations:** 1 Department of Biotechnology and Food Microbiology, Institute of Food Science Research (CIAL) (CSIC-UAM), 28001 Madrid, Spain; sara.lara.abia@gmail.com; 2 Tecnologico de Monterrey, Escuela de Ingeniería y Ciencias, Monterrey 64000, Mexico; jwelti@tec.mx

**Keywords:** high hydrostatic pressure, papaya fruits, *Carica papaya*, carotenoids, carotenoid esters, microstructure, INFOGEST^®^ digestion, stability, bioaccessibility

## Abstract

High hydrostatic pressure (HHP) is a non-thermal technology widely used in the industry to extend food shelf-life and it has been proven to enhance the extractability of secondary metabolites, such as carotenoids, in plant foods. In this study, fresh-cut papaya pulp of varieties (Sweet Mary, Alicia and Eksotika) from the Canary Islands (Spain) were submitted to the HHP process (pressure: 100, 350 and 600 MPa; time: come-up time (CUT) and 5 min) to evaluate, for the first time, individual carotenoid and carotenoid ester extractability and to assess their bioaccessibility using an in vitro simulated gastrointestinal digestion assay, following the standardized INFOGEST^®^ methodology. In addition, changes in papaya pulp microstructure after HHP treatments and during the different phases of the in vitro digestion were evaluated with optical light microscopy. HPLC-DAD (LC-MS/MS (APCI+)) analyses revealed that HHP treatments increased the carotenoid content, obtaining the highest extractability in pulp of the Sweet Mary papaya variety treated at 350 MPa during 5 min (4469 ± 124 μg/100 g fresh weight) which was an increase of 269% in respect to the HHP-untreated control sample. The highest carotenoid extraction value within each papaya variety among all HHP treatments was observed for (all-*E*)-lycopene, in a range of 98–1302 μg/100 g fresh weight (23–344%). Light micrographs of HHP-treated pulps showed many microstructural changes associated to carotenoid release related to the observed increase in their content. Carotenoids and carotenoid esters of papaya pulp submitted to in vitro digestion showed great stability; however, their bioaccessibility was very low due to the low content of fatty acids in papaya pulp necessary for the micellarization process. Further studies will be required to improve papaya carotenoid and carotenoid ester bioaccessibility.

## 1. Introduction

The relationship between diet and health has been demonstrated constantly over human history [1,2]. A healthy diet protects against non-communicable diseases, such as cardiovascular diseases, cancer, hypertension and obesity [3,4,5]. Fruit and vegetables contain a large amount of different dietary phytonutrients, which contribute to the prevention of diseases caused by oxidative stress. Numerous investigations have estimated that one-half of hypertension and cardiovascular diseases and one-third of cancer cases could be attributed to diet [6,7,8,9,10].

Among the most worldwide traded tropical fruits (mango, pineapple, avocado and papaya) papaya is the least commercialized, but its trade has grown promisingly over the past decade [11]. The United States of America (USA) is the main importer of papaya, with an estimated share of 70%, followed by the European Union, with a share of 15% in 2018 [12]. During 2015–2019, Mexican papaya production was about 984,000 tons, with an average annual growth rate of 5.2%. In 2020, the production of papaya in Mexico grew by 3.2% when totaling 1.11 million of tons, being the third papaya producer worldwide. During that year, Mexico was positioned as the main papaya exporter around the world with a participation volume of 44.7% and an annual growth rate of 2.4%. The latest data available indicate that the per capita availability of papaya in the USA stood at 0.6 kg. Despite the fact that the European Union ranks second largest importer, consumer awareness relative to this fruit is still low, with a per capita papaya consumption of 0.1 kg [12]. The promotion of this fruit and its nutritional benefits are, therefore, key to support the import and demand of papaya fruits, especially in the European Union. In Spain, the introduction in the market of three new papaya varieties, i.e., Sweet Mary, Alicia and Eksotika, cultivated in the Canary Islands, has caught the interest in our research group due to their interesting carotenoid profile based on a recent study reported by Lara-Abia et al. [13], where the utilization of these new varieties as a source of bioactive compounds is a real possibility for industrial applications.

Papaya (*Carica papaya* L.) is an important source of carotenoids, with β-carotene, β-cryptoxanthin, lutein and lycopene being the principal carotenoids found in this fruit [14,15]. Food carotenoids present several important biological activities, their provitamin A activity being the most studied activity related to human health. However, only those carotenoids with a β-ring in their structure, such as β-carotene and β-cryptoxanthin, are able to execute provitamin A activity. Vitamin A deficiency causes night blindness, xerophthalmia, skin irritation, keratinization, stunted growth, as well as weaking the immune system and compromising fertility, among other conditions. In developing countries, vitamin A deficiency is the major cause of children premature death [16]. Carotenoids also present biological functions of growth promoting, embryonal development, visual function and have protective effects against cancer. The intake of food rich in carotenoids scavenge free radicals, such as peroxide, hydroperoxide or lipid peroxyl, thus inhibiting the oxidative mechanism that leads to degenerative diseases [17,18].

Papaya fruits have been extensively studied due to their high consumption worldwide and because of the health benefits they promote. A recent study reported by Ramos-Parra et al. [19] concluded that high hydrostatic pressure (HHP) treatments on papaya cv. Maradol, significantly increased their carotenoid content after storage due to the oxidative stress derived after applying this technology, promoting de novo carotenoid biosynthesis. Hernandez-Brenes et al. [20] evaluated the effect of different HHP treatments on the extractability of carotenoids from papaya cv. Maradol purees. The authors studied three different pressure intensities (400, 500 and 600 MPa), at two processing times (1 and 3 min) and two processing temperatures (25 and 40 °C). They reported that total carotenoids were significantly (*p* > 0.05) affected by pressure and temperature, while the processing time did not have a significant (*p* < 0.05) effect on the extraction of carotenoids, obtaining around of 154% higher content of total carotenoids in papaya puree processed at 600 MPa/25 °C/3 min than that yielded from the untreated samples. Likewise, Cano et al. [21] evaluated the effect of HHP treatment (200 MPa/25 °C/6 min) and pasteurization (85 °C/15 min) on carotenoid stability and bioaccessibility of carotenoids and carotenoid esters from astringent persimmon fruits (*Diospyros kaki* Thunb., var Rojo Brillante). As reported for papaya fruits, these authors observed an increase in the concentration of carotenoids after HHP treatment compared with the pasteurization process and the untreated samples. In addition, they observed that pressurized samples showed higher bioaccessibility than pasteurized samples. The differences in the bioaccessibility could be associated to the effect of each treatment on the pectin present in persimmon tissues. The characteristics of pectin, i.e., methylation degree, and the modifications generated by the processing technology used influence bioaccessibility hindering carotenoid intestinal absorption. Even though papaya fruits from different cultivars have been previously investigated and their phytochemical and nutritional characteristics have been reported, there is a lack of information concerning the effect of non-thermal technologies, i.e., HHP, on individual carotenoid and carotenoid ester content in papaya pulp (edible tissue) and in their stability and bioaccessibility during in vitro gastrointestinal digestion. 

The bioactivity of carotenoids not only depends on their chemical structure, but also on their absorption and metabolism in the human body [22]. In the last years, numerous articles have revealed the relationship between carotenoid bioavailability and bioaccessibility, and health. Through the cooperation of the International Network of Excellence on the Fate of Food in the Gastrointestinal Tract (INFOGEST) a standardized in vitro digestion protocol was established in order to harmonize methodologies to determine the effect of food matrices on the bioavailability of food nutrients and bioactive molecules during the digestion process. Rodrigues et al. [23] reported an in vitro digestion protocol adapted for carotenoids and carotenoid esters to improve bioaccessibility studies of these lipophilic compounds. The absorption of carotenoids and other lipophilic compounds depends, first, on their release from the food matrix through digestion and their incorporation into bile salt mixed micelles in the intestinal lumen. Not all carotenoids present the same uptake in the human body; oxygenated carotenoids (xanthophylls and xanthophyll esters) are more easily incorporated into the lipid micelles in the gastrointestinal tract than hydrocarbon carotenoids due to their different polarity [24,25]. However, it is not only the polarity that interferes in the absorption of carotenoids; other factors, such as the food matrix, the amount and type of fat an dietary fiber and how the product is processed, may affect their absorption as well. Schweiggert et al. [25] also pointed out that the different deposition of carotenoids in the chromoplasts within the vegetable cells also influenced carotenoid liberation efficiency from the food matrix.

Food processing has changed in the last decades. Traditionally, conventional techniques, such as pasteurization and sterilization, were used to preserve food stability, but thermal processing presents some disadvantages over food quality, such as destroying nutrients, off-flavor formation and discoloration, among other undesired effects [26,27]. HHP processing has been an accepted alternative due to its limited effects over covalent bonds, which minimizes modifications in sensorial and nutritional food quality [28,29]. Up to now, there are numerous studies about the effectiveness of HHP in destroying microorganisms in fruits and vegetables; further, some studies have suggested [26,29] that this non-thermal technology may enhance antioxidant activity and improve the extraction of bioactive compounds due to the changes that occur in plant food structure during food processing. 

The objective of this study is to evaluate the possibility to enhance the papaya carotenoid and carotenoid ester extractability by HHP-assisted extraction. In addition, the stability and bioaccessibility of carotenoids and carotenoid esters in the pulp of the Sweet Mary papaya variety were studied using the INFOGEST^®^ methodology to assess the simulated in vitro gastrointestinal digestion. Additionally, the effect of HHP treatments on the papaya pulp structure was evaluated to study the localization of carotenoids and their possible release from the cell organelles after processing and in the different phases of the in vitro gastrointestinal digestion process.

## 2. Materials and Methods

### 2.1. Plant Material

The papaya (*Carica papaya* L., cv. Sweet Mary, Alicia and Eksotika) fruits used in the study were obtained from the Spanish region of Tenerife (Canary Islands, Spain: 28°18′52° north (N); 16°24′36° west (W); 271 m above sea level). Papaya fruits were washed and selected according to uniform size and maturity [30]. No effects were detected in the selected fruit pieces. Fruits physical-chemical characteristics were evaluated as described previously by Plaza et al. [31] and they are detailed in Table S1, Supplementary Materials.

### 2.2. Chemicals, Standards and Reagents

Milli-Q water was obtained from a Millipark^®^ Express 40 system (Merk-Millipore, Darmstadt, Germany); methanol, diethyl ether, tetrahydrofuran (THF), methyl tert-butyl ether (MTBE), acetone and petroleum ether were purchased from VWR International (Radnor, PA, USA). Butylated hydroxytoluene (BHT) and magnesium carbonate were obtained from Acros Organics (New Jersey, USA). Anhydrous sodium sulphate, sodium chloride (NaCl) and potassium hydroxide (KOH) were purchased from Panreac Quimica (Barcelona, Spain). Standards for lycopene (L9879, ≥90%, from tomato), (all-*E*)-β-apo-8′-carotenal (10810, ≥96%, (UV)) and lutein (X6250 from marigold) were purchased from Sigma-Aldrich (St. Louis, MO, USA). Standards for (all-*E*)-β-carotene (HPLC 96%, synth., cryst.), (all-*E*)-α-carotene (HPLC 97%, synth., cryst.) and (all-*E*)-β-cryptoxanthin (HPLC 97%, synth., cryst.), (all-*E*)-violaxanthin (HPLC 95%, isolated, cryst.), (all-*E*)-neoxanthin (HPLC 97%, isolated, cryst.) and (all-*E*)-zeaxanthin (HPLC 97%, synth., cryst.), were purchased from CaroteNature (Ostermundigen, Switzerland). Enzymes used in the simulated gastrointestinal in vitro digestion were acquired from Sigma-Aldrich (St. Louis, MO, USA). The following enzymes were used: α-amylase from porcine pancreas (10080; 79 U mg^−1^), pepsin from porcine gastric mucosa (P6887; 791 U mg^−1^), pancreatin from porcine pancreas (P7545; 17 units TAME per mg), bile salts from bovine and ovine origin (B8381). Other reagents used in the in vitro digestion assay were acquired from Sigma-Aldrich (St. Louis, MO, USA).

### 2.3. High Hydrostatic Pressure Treatments

Ten fully mature fruits of each papaya variety (Sweet Mary, Alicia and Eksotika) were selected based on size, weight and full peel coloration (Table S1, Supplementary Materials). Selected fruits were washed and peeled and the seeds were removed. Then, they were sliced manually in uniform cubes (~1 cm^3^) and placed in food-grade Doypack^®^ bags (VWR International, Barcelona, Spain) which were vacuum-sealed for each treatment. Fruits were pressurized in a HHP equipment (2 L capacity, Stansted SFP 7100:9/2C, UK). The pressure-transmitting medium was Blue Sun^®^ (Capermar SL, Zaragoza, Spain) diluted in water (20%, v/v). HHP treatment conditions were pressures of 100, 350 and 600 MPa and come-up time (CUT) and holding time (HT) of 5 min were applied. CUT was obtained by pressurizing samples until the target pressure was reached and held for 1 s before decompression. The processing average temperature was 26.2 ± 1.8 °C, the compression rate was 2.6 MPa/s and decompression occurred almost instantly (<1 s). The CUT for 100, 350 and 600 MPa treatments were 61, 156 and 209 s, respectively. Pressure, time and temperature were constantly controlled, monitored and recorded throughout the process by a computer program. The average of HHP parameters is detailed in Table S2 (Supplementary Materials). Each HHP treatment was performed twice. After pressurization, papaya samples were immediately frozen with liquid nitrogen. These frozen samples were freeze-dried for 5 days at −45 °C and 1.3 × 10^−3^ MPa (LyoBeta 15, Azbil Telsar SL, Terrasa, Spain). Freeze-dried material was stored at −80 °C until the HPLC analyses of the individual carotenoids and carotenoid esters to conduct the study on the effect of HHP treatments on the extraction of carotenoids in the three varieties of papaya. After carotenoid analyses, the papaya variety that showed the highest carotenoid concentration (cv. Sweet Mary) after pressurization was selected to follow the simulated gastrointestinal in vitro digestion procedure, as mentioned in the section below. Papaya samples frozen with liquid nitrogen and stored at −80 °C were also used for the microscopy studies.

### 2.4. In Vitro Gastrointestinal Digestion Assay

The in vitro gastrointestinal digestion assay of papaya fruits cv. Sweet Mary was performed according to the standardized INFOGEST© protocol [32] with the adaptations for carotenoid studies reported by Cano et al. [21]. Digestive solutions for mouth (simulated saliva fluid, SSF), stomach (simulated gastric fluid, SGF) and small intestinal (simulated duodenal fluid, SDF) compartments were prepared following the methodology described by Eriksen et al. [33]. To avoid loss of activity and denaturalization, enzymes solutions were prepared daily prior to the digestive assay. After each phase (oral, gastric and intestinal) of the in vitro digestion, a digestive sample was obtained, named digesta. The digesta was ultra-centrifuged (L-70 Ultracentrifuge Beckman Coulter, CA, USA) at 20,000× *g* for 10 min at 4 °C. The liquid was referred to as supernatant, while the pellet was discarded. Carotenoid bioaccessibility was calculated as the ratio between carotenoid concentration in the supernatant of the intestinal fraction and its initial concentration in the fruit, this value corresponding to the concentration in the control sample and in the HHP treated samples prior to in vitro digestion in each case (Equation (1)).

Bioaccessibility (%) = (Carotenoid content_supernatant_/Carotenoid content_fruit tissue_) × 100 (1)

### 2.5. Carotenoid Analysis

#### 2.5.1. Carotenoid Extraction from Fresh and Freeze-Dried Papaya

The extraction of carotenoids and carotenoid esters was performed following Cano et al.’s [21] methodology with modifications. First, 5 g of fresh papaya pulp or 1 g of freeze-dried papaya pulp was weighed. Then, 0.5 g of magnesium carbonate, 60 mL of (all-*E*)-β-apo-8′-carotenal (internal standard) (0.40 mg/mL) and 20 mL of tetrahydrofuran (THF) stabilized with 0.01% (w/v) of butylated hydroxytoluene (BHT) were added. The sample was homogenized in an Omnimixer (OMNI Macro ES^®^, OMNI International, Kennesaw, GA, USA) for 3 min at 7000 rpm and placed in an ultrasonic water bath (3000514 model, 50/60 Hz, 360 W, J. P. Selecta S.A., Barcelona, Spain) for 30 min. The extract was centrifuged at 15,000× *g* for 10 min at 4 °C and the supernatant was collected. The extraction with 20 mL of TFH (with BHT 0.01% v/v) was repeated two more times, leaving the sample in the ultrasonic water bath 15 min each time. Then, 20 mL of methanol was added to the pellet and the sample was extracted again. The supernatants of each extraction were combined and placed in a separation funnel. After, 20 mL of diethyl ether were added to the funnel, which was shaken vigorously and the organic layer was collected and dried with 2.5 g of anhydrous sodium sulphate for 10 min at room temperature under dim light. Then, it was filtered and dried at 30 °C in a rotavapor, made up to 2 mL with MeOH/MTBE/H2O (45.5:52.5:2, v/v/v). Finally, the sample was filtered through a 0.45 μm filter and analyzed by HPLC. In addition, a duplicate of carotenoid extracts of each sample was saponified by adding 7 mL of 30% KOH in methanol under nitrogen atmosphere and darkness for 1.5 h following previously reported methodology [34].

#### 2.5.2. Carotenoid Extraction from In Vitro Digestion Phases

Carotenoid extraction from the digesta was performed according to de Petry and Mercadante [35] with modifications. The digesta was extracted with 15 mL of acetone and placed in an ultrasound bath for 15 min at 25 °C. Then, samples were centrifuged at 10,000× *g* for 10 min at 4 °C and the supernatant was recovered. The pellet was re-extracted twice with acetone and one last time with methanol. Afterwards, in a separation funnel, 30 mL of petroleum ether/diethyl ether (1:1; v/v) was added to the combined supernatants. The organic phase was recovered and dried with anhydrous sodium sulphate (approx. 2.5 g), filtered and evaporated until dry in a rotavapor at 30 °C. The sample was made up to 2 mL with MeOH: MTBE: H2O (45.5:52.5:2, v/v/v), filtered through a 0.45 μm filter and analyzed by HPLC. 

Carotenoid extraction from the supernatants of the digestive phases was performed according to Petry and Mercadante [35] with modifications. Each supernatant, obtained after ultra-centrifugation, was extracted with 20 mL of diethyl ether by mixing with a vortex and centrifuging at 14000 rpm for 10 min at 4 °C in an L-70 Ultracentrifuge (Beckman Coulter, Indianapolis, IN, USA). Then, the supernatant (micellar) fraction was placed in a separation funnel and 20 mL of diethyl ether was added and shaken vigorously. The organic phases were recovered, dried with anhydrous sodium sulfate, filtered and vacuum-concentrated to be analyzed by HPLC.

### 2.6. Carotenoid Analysis by HPLC-DAD

The analysis of carotenoids and carotenoid esters was performed following the methodology previously described by Cano et al. [21] using a reversed phase C_30_ column (YMC-Pack YMC C_30_, 250 × 4.6 mm i.d., S-5 μm, YMC Co., Ltd. (Kyoto, Japan) and a 1200 Series Agilent HPLC System) Agilent Technologies, Santa Clara, CA, USA). The mobile phase was methanol: MTBE: water (81:140:40, v/v/v, eluent A) and methanol: MTBE (10:90, v/v, eluent B), both containing 0.1% of ammonium acetate. The elution gradient was linear, starting at 100% A and ending with 100% B, in 60 min. Carotenoids were detected at 450 nm. Additional UV/Vis spectra were recorded between 220–700 nm. Carotenoids were identified based on their elution time in the column, UV-Vis spectra (λmax, peak cis intensity and spectral fine structure (% III/II)), by comparison with carotenoid standards and mass spectrum compared with available data [21,36,37,38,39,40]. 

Carotenoid quantification was carried out with carotenoid calibration curves (concentration range of 5–100 μg/mL of carotenoid stock solution). The (all-*E*)-lycopene curve was used for quantifying lycopene and lycopene isomers; for the quantification of (all-*E*)-β-carotene, (all-*E*)-α-carotene and their isomers, the (all-*E*)-β-carotene and (all-*E*)-α-carotene calibration curves were used, respectively. Carotenoids such as (all-*E*)-violaxanthin and (all-*E*)-neoxanthin were quantified with their corresponding standards. Contents of α-cryptoxanthin and β-cryptoxanthin and their esters were calculated on the basis of the (all-*E*)-β-cryptoxanthin calibration curve. The (all-*E*)-lutein calibration curve was used for lutein-epoxide and lutein ester quantification. The (all-*E*)-violaxanthin standard curve was used to quantify (all-*E*)-violaxanthin, violaxanthin isomers and (all-*E*)-antheranxanthin. Vitamin A value was calculated as retinol activity equivalent (RAE) per 100 g of fresh weight, following the equation RAE = (μg of β-carotene/12) + (μg of other pro-vitamin A carotenoids, such as β-cryptoxanthin and β-cryptoxanthin esters)/24) (Institute of Medicine (US) [41]). Results were expressed as micrograms of the corresponding carotenoid per 100 g of fresh weight. Carotenoid esters were quantified using the calibration curves of their corresponding carotenoids.

### 2.7. Liquid Chromatography Mass Spectrometry (LC-MS/MS (APCI^+^))

The LC-MS/MS (APCI^+^) analyses were performed using HPLC coupled to a mass spectrometry detector with an APCI source model G1947B (Agilent) compatible with LCMS SQ 6120 equipment [42]. Nitrogen was used as the drying gas at a flow rate of 60 L/min and as nebulizing gas at a pressure of 50 psi. The nebulizer temperature was 350 °C and, on the capillary, a potential of +2779 kV was used. Helium was the collision gas and the fragmentation amplitude was of 0.8–1.2 V. The vaporizer temperature was set at 400 °C and the corona was 4000 nA as positive ion mode. The positive ion mass spectra of the column eluted at 13,000 Th/s (peak width 0.6 Th, FWHM).

### 2.8. Optical Microscopy

Optical microscopy was used for the analysis of carotenoids in papaya pulp tissue and to study the microstructural changes produced by HHP treatments in fruit pulp of the different papaya varieties studied. Cryostat sections (20 μm) were obtained from fresh papaya cubes and cubes that had just been HHP-processed (immediately after pressurization). Papaya pulp samples were frozen (−80 °C), mounted on a cryostat (Leica CM1900) and cut in slices with a monitored microtome (Leica RM2155). Several drops of the digesta of each digestion (oral, gastric and intestinal) phases were placed on a crystal slice and observed in the microscope, to observe the carotenoids in the different phases of the in vitro gastrointestinal digestion of papaya cv. Sweet Mary pulp HHP-treated and control samples. No dye was used to stain the samples. The optical microscopy study was performed with a vertical microscope Axioskop (Carl Zeiss, Jena, Germany) coupled to a Leica DMC 6200 pixel shift camera (Leica Microsystems, Wetzlar, Germany). Samples were observed with no color filters, an open condenser and level 4 of illumination. The color of the image was manually adjusted to reflect real-time colors (0.90 gamma, 45% saturation and 65% brightness) using the Leica Application Suite software. Samples were observed at 20× and at 40× with a Zeiss Plan-Neofluar lens. At least three replicas of each sample were prepared and analyzed.

### 2.9. Statistical Analysis

The results were analyzed by an analysis of variance (ANOVA) and significant differences were calculated by Tukey‘s b test (*p* ≤ 0.05). The data are expressed as mean ± standard deviation. In vitro gastrointestinal digestions were carried out at least twice for each sample and all analyses were performed three times. The statistical analyses were performed using the SPSS software (version 20.0, SPSS Statistical Software, Inc., Chicago, IL, USA).

## 3. Results and Discussion

### 3.1. Characterization of Carotenoid and Carotenoid Ester Profile

#### 3.1.1. Individual Carotenoid Profile in Papaya Tissues

The identification and quantification of carotenoids and carotenoid esters on the pulp tissue of the Sweet Mary, Alicia and Eksotika papaya varieties has been previously reported by Lara-Abia et al. [13]. The results reported in the mentioned investigation were considered as the control data for this study due to the simultaneity of the two studies. Total carotenoid contents in unsaponified papaya pulp extracts, as sum of individual carotenoids, were 1664 ± 49, 1595 ± 40 and 2148 ± 64 μg/100 g fresh weight in Sweet Mary, Alicia and Eksotika varieties, respectively. In addition, in that study, carotenoid content in Maradol papaya tissues was reported. In unsaponified pulp papaya cv. Maradol extracts, carotenoid content was 3908 ± 84 μg/100 g fresh weight. Similar data concerning the Maradol papaya variety have been already published by other authors [20,25]. In the present study, unsaponified extracts obtained from papaya pulps treated with HHP were the main focus of the performed analyses. However, the individual carotenoid content in the extracts was also analyzed in order to complete the information.

The identification and quantification of carotenoids and carotenoid esters on the pulp tissue of the Sweet Mary, Alicia and Eksotika papaya varieties has been previously reported by Lara-Abia et al. [13]. The results reported in the mentioned investigation were considered as the control data for this study due to the simultaneity of the two studies. Total carotenoid contents in unsaponified papaya pulp extracts, as sum of individual carotenoids, were 1664 ± 49, 1595 ± 40 and 2148 ± 64 μg/100 g fresh weight in Sweet Mary, Alicia and Eksotika varieties, respectively. In addition, in that study, carotenoid content in Maradol papaya tissues was reported. In unsaponified pulp papaya cv. Maradol extracts, carotenoid content was 3908 ± 84 μg/100 g fresh weight. Similar data concerning the Maradol papaya variety have been already published by other authors [20,25]. In the present study, unsaponified extracts obtained from papaya pulps treated with HHP were the main focus of the performed analyses. However, the individual carotenoid content in the extracts was also analyzed in order to complete the information.

#### 3.1.2. Individual Carotenoid and Carotenoid Ester Content in Papaya Pulp Processed by HHP

In the HHP study reported herein, three different pressure intensities (100, 350 and 600 MPa) were applied at two different times (CUT and 5 min) with constant temperature (25 °C). Results indicated that HHP treatments did not influence the chromatographic profiles of carotenoids in the papaya pulp (Figure S1) of the three studied varieties. However, individual quantitative changes in carotenoids induced by pressure level and time after the application of HHP treatments were observed (data of the individual carotenoid content are shown in Table 1 for cv. Sweet Mary, in Table S3 for cv. Alicia and in Table S4 for cv. Eksotika). Statistically, significant differences (*p* ≤ 0.05) were found between treatments within each papaya variety. However, no significant differences were observed when applying 100 MPa during the CUT and 5 min for the Sweet Mary and Alicia papaya varieties; further, in the Alicia variety, no significant differences were found after the 350 MPa/5 min and 600 MPa/5 min treatments. A total of 46 different carotenoids were identified and quantified after HHP treatments, of which 16 were free xanthophylls, 14 hydrocarbon carotenoids and 16 xanthophyll esters. Figure S2 (Supplementary Materials) shows the carotenoid content distributed on the different classes of carotenoids, total hydrocarbon carotenoids, total free xanthophylls and total xanthophyll esters, in direct (unsaponified) extracts of the papaya pulps of the three studied varieties, submitted to HHP treatments. In addition, to complement this information, in Figure S3 (Supplementary Materials) the carotenoid content in the saponified extracts of the HHP treated pulps is presented. Among all individual carotenoids, (all-*E*)-lycopene showed the highest concentrations with a range of 98–1302 μg/100 g fresh weight, followed by (all-*E*)-β-carotene (51–649 μg/100 g fresh weight), (all-*E*)-β-cryptoxanthin (21–388 μg/100 g fresh weight) and some β-cryptoxanthin esters, such as β-cryptoxanthin caprate (20–236 μg/100 g fresh weight) and laurate (41–365 μg/100 g fresh weight) within all HHP treated pulp samples of the three papaya varieties. Total carotenoid content in Sweet Mary treated pulps, showed the highest content, compared to Alicia and Eksotika varieties, as shown in Table 1. Sweet Mary pulp treated at 350 MPa during 5 min showed the highest total carotenoid content (4469 ± 124 μg/100 g fresh weight); in contrast, applying the same pressure during the come-up time (CUT) the total carotenoid content was 2841 ± 89 μg/100 g fresh weight). When the pressure applied was 100 MPa for CUT and 5 min time, the carotenoid content did not show any significant differences (1807 ± 11 μg/100 g fresh weight and 2023 ± 15 μg/100 g fresh weight, respectively) in respect to the correspondent control sample. On the other hand, Sweet Mary pulp samples treated at 600 MPa/CUT produced the highest total carotenoid content (2336 ± 33 μg/100 g fresh weight), compared to the application of the same pressure for 5 min process time (1496 ± 99 μg/100 g fresh weight), whose carotenoid extraction was the lowest compared to the other HHP treatments. Although the temperature of the pressurization medium was constantly monitored during each treatment (26.2 ± 1.8 °C), the adiabatic heat related to pressure leads to temperature increasing in the product treated, which may vary depending on food composition (fluctuating from 3 °C/100 MPa in foods with high moisture, as papaya fruit pulp, to 8 °C/100 MPa in fruits with high fat composition) [43]. Thus, product temperature may imply a higher thermal effect associated with the HHP in food matrices at higher pressure levels. The processing carried out at 600 MPa could be generating the greatest increase in the temperature of the treated samples and, as a consequence, inducing greater thermal degradation of carotenoids regarding the decrease in total carotenoid content. In addition, it has been reported that pressures levels in the range of 10–200 MPa might cause stress without compromising cell integrity, while, above 200 MPa, they are more likely to induce damage and cell death in plant tissue [44]. Ramos-Parra et al. [19] reported that HHP treatments caused an increase in carotenoid content in papaya pulps treated at different levels of pressure, indicating that HHP treatments cause oxidative stress on the plant cell that triggers the production of carotenoids in papaya fruits due to the increase of de novo carotenoid biosynthesis at transcriptional level by modulating the expression of genes involved in carotenogenesis. Plaza et al. [31] reported that the HHP treatment at 200 MPa/25 °C/6 min of astringent persimmon samples showed an increase in carotenoid extractability in regard to the control sample. In another persimmon study reported by Vazquez-Gutierrez et al. [45], it was concluded that HHP treatments favored the extractability of soluble compounds, e.g., tannins and their diffusion into the intercellular spaces due to changes in cohesiveness and firmness of the treated samples. Jacobo-Velazquez et al. [46] concluded that applying 600 MPa during 3 min caused a significant increase (56% approximately) in the extraction of carotenoids from avocado paste. Hernandez-Carrion et al. [47] reported that the effect of HHP treatments (100 MPa, 200 MPa, 300 MPa and 500 MPa at 25 °C for 15 min) caused a statistically significant reduction (*p* < 0.05) in the carotenoid content of sweet peppers in contrast to the non-treated samples. On the other hand, several studies have not reported significant differences (*p* > 0.05) on carotenoid content between HHP treated samples and control using different plant product matrices, such as papaya beverages [48,49], mango nectars [29] and oranges juices [50]. As it appears, due to the general view, the effect of HHP treatments on the carotenoid content is related to the plant material to which this technology is applied; therefore, no general conclusions can be drawn.

According to the results obtained in this part of the investigation, the Sweet Mary variety was selected to perform the study of the stability and bioaccessibility of individual carotenoids and carotenoid esters in control and HHP-treated pulps by in vitro gastrointestinal digestion. The high carotenoid content of this papaya variety was obtained after applying HHP, especially regarding the content of (all-*E*)-lutein, (all-*E*)-β-cryptoxanthin and their esters, in comparison with the Alicia and Eksotika papaya varieties (Tables S3 and S4, Supplementary Materials).

### 3.2. Stability of Carotenoids and Carotenoid Esters in Papaya during In Vitro Digestion

**Figure 1:**
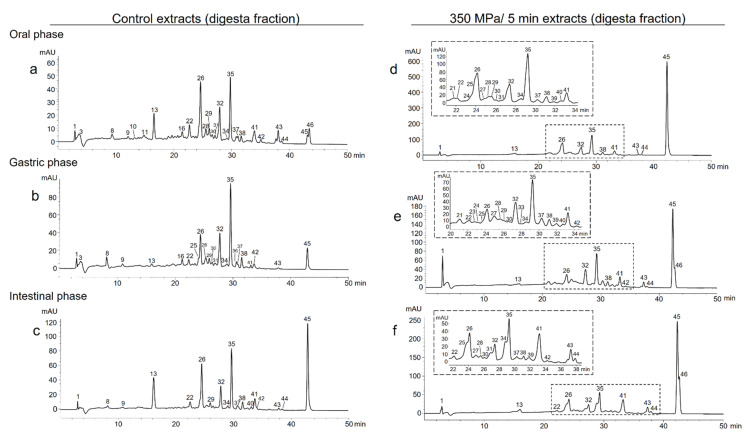
C_30_ reversed-phase chromatograms of carotenoids at 450 nm obtained from cv. Sweet Mary papaya (*Carica papaya* L.) pulp after in vitro gastrointestinal digestion (digesta fraction) of untreated (control) sample—(**a**) oral phase; (**b**) gastric phase; (**c**) intestinal phase—and submitted to HHP (350 MPa/5 min) sample—(**d**) oral phase; (**e**) gastric phase; (**f**) intestinal phase. Peak identities in Table 1.

C_30_ reversed-phase chromatograms of carotenoids at 450 nm from Sweet Mary pulp direct extracts after in vitro gastrointestinal digestion (digesta fraction) previously treated at 350 MPa/5 min and from the untreated sample (control) are presented in Figure 1. The corresponding chromatograms of their micellar fractions are shown in Figure S4, Supplementary Materials. The data of the carotenoid content in Sweet Mary papaya pulp submitted to HHP treatments after each phase of in vitro digestion are presented in Table S5 (Supplementary Materials). In order to have a wider vision about the effects of HHP treatments on the extraction of carotenoids from papaya pulp (cv. Sweet Mary) after in vitro digestion, the recovery (%) of the individual carotenoids identify on each digestion phase, along with total free xanthophylls, total xanthophyll esters and total hydrocarbon carotenoids, is shown in Table 2. The carotenoid recovery is the relation among the carotenoid content in the digesta in each phase of the in vitro digestion and the carotenoid content in the undigested sample (before in vitro digestion) [35].

Total carotenoid recovery in the oral phase digesta of the treated Sweet Mary papaya pulp ranged from 22.0 to 84.6%, depending on the studied carotenoid, while, in the untreated control papaya pulp, a total carotenoid recovery of 23.4% was observed in this oral phase (Table 2). Papaya pulp had a wide variety of xanthophyll esters, which showed a different release determined by the hydrolysis of the α-D-(1-4)-glycosidic linkages of the pectin and other similar compounds present in papaya cells, over which the α-amylase, added in the oral phase of the in vitro digestion, executed its action. During the oral phase of the digestion, the xanthophyll ester recoveries from the digesta were relatively high (44.0–73.0%) except for samples treated at 100 MPa/5 min and 350 MPa/CUT, which showed lower recovery of xanthophyll esters (4.8 and 38.2%, respectively) than the untreated (control) sample (42.3%). (9Z)-violaxanthin dimyristate also showed high recoveries (55.0–108.3%) and a great stability in all HHP treated pulp samples (except for 100 MPa/5 min). In addition, in the untreated sample, a recovery of 41.1% was obtained for this carotenoid ester, that, compared to the rest of the xanthophyll esters found in papaya pulp, showed to be more unstable and/or had lower recoveries when a general analysis was performed. According to Petry and Mercadante [35], (*Z*)-carotenoid esters are more likely to be hydrolyzed than their respective (all-*E*)-isomers regarding the concentration of the bile salts used in the in vitro digestion. However, this hypothesis must be confirmed with further studies. In the oral phase, the recovery of total hydrocarbon carotenoids found in control pulp samples was similar to the one observed for 100 MPa/CUT (39.6%) and 100 MPa/5 min (33.3%) and for 350 MPa/CUT (46.4%) treated samples. The recovery at 350 MPa/5 min was 72.5% and the HHP treatment at 600 MPa/5 min produced a recovery of 83.1% of the total hydrocarbon carotenoids, which was higher than the observed for control sample (34.2%), while the papaya pulp samples treated at 600 MPa/CUT showed the highest recovery (89.2%) of these carotenoids.

**Figure 2:**
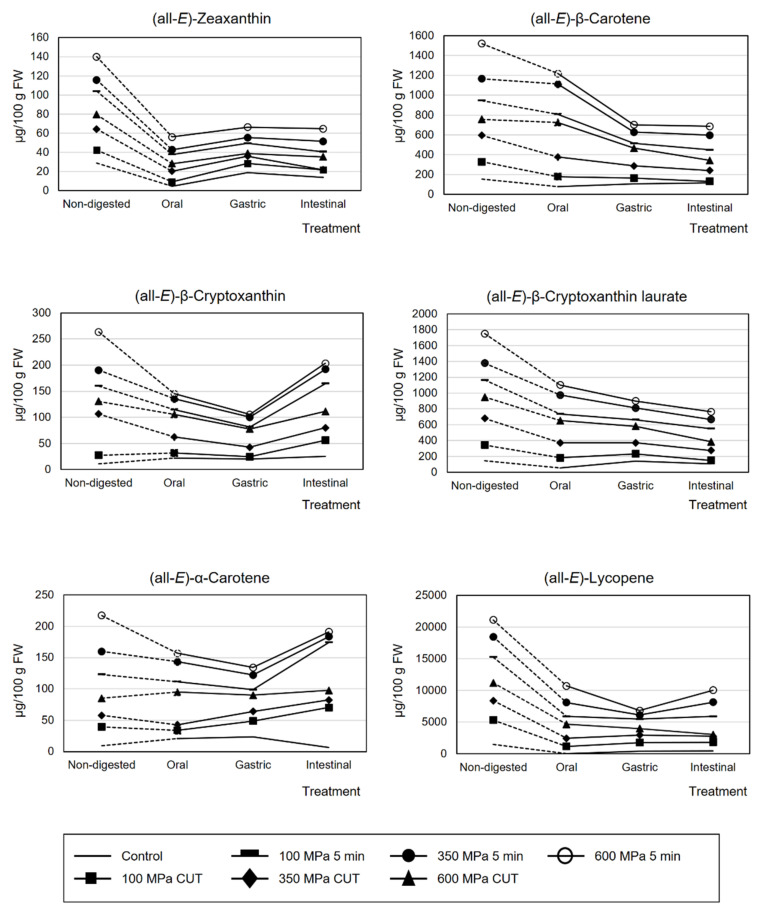
Bioactive content (μg carotenoids/100 g fresh weight) of (all-*E*)-zeaxanthin, (all-*E*)-β-cryptoxanthin, (all-*E*)-α-carotene, (all-*E*)-β-carotene, (all-*E*)-β-cryptoxanthin laurate and (all-*E*)-lycopene in papaya (*Carica papaya* L.) cv. Sweet Mary pulps submitted to HHP treatments after in vitro gastrointestinal digestion.

In the gastric phase, the activity of the pepsin and the acid environment created by hydrochloric acid (HCl) stimulated the separation between the fatty acids and the xanthophylls in the xanthophyll esters by a hydrolysis process. Therefore, an increment of free xanthophylls recovery was observed in all HHP papaya pulp treated samples (Table 2). The recovery of (all-*E*)-violaxanthin ranged from 39.6 to 181.9%, with 175.4% being the recovery obtained from the untreated pulp sample. In contrast, this increment of recovery was not observed in all HHP pulp treated samples for (all-*E*)-zeaxanthin, showing that treatments at 350 MPa/CUT, 600 MPa/CUT and 600 MPa/5 min have an opposite tendency to that the observed for (all-*E*)-violaxanthin in these gastric phases. Only traces of (all-*E*)-antheranxanthin were found in HHP pulp samples during in vitro digestion, except for samples treated at 100 MPa/CUT and 600 MPa/5 min, with the low content of 7.5 and 8.2 μg/100 g of fresh weight, respectively (Table S5). On the other hand, the free form of (all-*E*)-β-cryptoxanthin was identified in all HHP-treated papaya pulp samples (Figure 2) showing recovery values ranging from 6.9 to 144.9% (control sample recovery: 181.0%). In this case, it seems that the effect of high pressures affected the content of the non-esterified (all-*E*)-β-cryptoxanthin due to the observed significant differences found among the untreated sample (Table S5, Supplementary Materials). The pulp sample treated at 600 MPa/CUT showed the highest total xanthophyll esters recovery (50.8%), compared to the rest of the HHP-treated samples. Petry and Mercadante [35] reported a decrease in (all-*E*)-β-cryptoxanthin ester content from mandarin pulp of 20–21% in the gastric phase and 5–6% in the intestinal phase, as a result of partial hydrolysis of carotenoid esters, which increased the free β-cryptoxanthin during digestion. It has been reported that, in human plasma, the content of free xanthophylls is higher than their esters after ingesting both at the same time. This fact suggest that carotenoid ester hydrolysis occurs prior to absorption [51].

The stability of the main carotenoids found in papaya HHP-treated pulps after in vitro gastrointestinal digestion is presented in Figure 2. Raw data may be consulted in Table S5. The stability of (all-*E*)-zeaxanthin in the intestinal phase ranged from 22.0 to 93.3% in the HHP treated samples and 47.6% of stability in the non-treated sample.

(all-*E*)-β-cryptoxanthin, the other abundant free xanthophyll in the digested phases, showed a stability range of 30.5–185.1%, slightly lower than the one observed for the untreated sample (232.2%) in the intestinal phase. Therefore, an HHP treatment of 100 MPa/CUT spared the greatest stability in free xanthophylls among all treated pulp samples. (all-*E*)-β-cryptoxanthin laurate, one of the most representative xanthophyll esters in papaya pulp, showed a stability range of 23.3–76.9%, very similar to the stability observed in the untreated sample (72.0%) in this digestion intestinal phase. Regarding (all-*E*)-β-carotene and (all-*E*)-lycopene, the two hydrocarbon carotenoids more abundant in papaya pulp, the recovery ranges were 25.1–66.9% and 10.0–30.4%, respectively, for HHP-treated samples. In addition, the stability values of (all-*E*)-β-carotene and (all-*E*)-lycopene in the intestinal phase of the in vitro digestion of the untreated samples, were 76.9% and 31.4%, respectively (Table 2 and Figure 2). According to Gomez-Maqueo et al. [52] in lucuma fruits, an observed high stability of these hydrocarbon carotenoids during in vitro digestion (62–63%) was directly related to the reduction of carotenoid liberation in the gastrointestinal process. In the present investigation, overall carotenoid and carotenoid ester stability in untreated papaya pulp showed 70.2% of stability, slightly higher than the overall stability observed for HHP-treated pulps (48.9–68.2%). These data agree with the stability data reported in fruits with high pectin content, such as persimmon (74%) [21]. Similar total carotenoid stability was observed in mandarin pulps (77%) [35] and in functional beverages composed of mango, papaya and açai juices mixed with orange juice and oats [49]. Recently, Laurora et al. [14] reported the carotenoid composition and bioaccessibility of papaya cultivars from Hawaii (yellow-fleshed papayas, Kapoho Solo, Lāʻie Gold/Kamiya and Rainbow; red-fleshed papayas, Sunrise and Sunset). In their investigation, although the overall carotenoid stability was not presented, they observed that the lycopene digestive stability was 55% in red-fleshed papayas, which was higher than the stability observed in the untreated papaya sample (31.4%) in the present study of the Sweet Mary variety and in HHP-treated pulp samples (10.0–71.1%).

The application of HHP improved the extraction of carotenoids and carotenoid esters in Sweet Mary papaya pulp but may also have affected their stability during gastric and intestinal phases, which could also be highly related to the presence of dietary fiber in papaya pulp. Papaya pulp has a dietary fiber content of 0.7–1.0%, which is higher than the fiber content in tomato (0.2–0.6%) [53], hindering the carotenoid release. Even though high hydrostatic pressure improved carotenoid release (extractability) from the food matrix, barely noticeable results in carotenoid recovery after in vitro digestion of pressure treated papaya pulp samples were observed in the present work. This fact could be related precisely to the dietary fiber, mainly in the presence of pectin, that modifies the stability and recovery of the carotenoids. A possible solution would be removing the pectin prior to in vitro digestion to avoid forming gels, decreasing the aqueous medium and allowing better carotenoid micellarization to occur. Further research is required to elucidate the interactions between particles and components in the food matrix during the digestion process.

Regarding total carotenoid recovery in the final phase (intestinal) of the in vitro simulated gastrointestinal digestion, only the treatments at 350 MPa/5 min and 600 MPa/5 min produced higher carotenoid recoveries (66.6% and 52.6%, respectively) in respect to the untreated sample (45.1%), indicating that high hydrostatic pressure treatment (HHP) did not have strong influence over carotenoid stability during in vitro digestion.

In the intestinal phase, more differences due to HHP treatments on carotenoid recoveries could be observed than in the oral and gastric phases, mainly in xanthophyll esters and in hydrocarbon carotenoids (Table 2). In the intestinal digesta, the untreated pulp sample showed higher total free xanthophylls recovery (132.8%) than in the oral digesta (93.5%) but not higher than the gastric digesta (160.5%). (all-*E*)-violaxanthin, (all-*E*)-zeaxanthin, (all-*E*)-antheraxanthin and (all-*E*)-β-cryptoxanthin showed recoveries of 150.8, 47.6, 129.4 and 232.2% in the untreated sample, respectively. The recovery of total free xanthophylls in the treated samples showed lower recovery range (27.6–107.0%) than the untreated sample (132.8%). Although saliva and gastric enzymes are less specific to release fatty acids than intestinal lipases, their activity also enhanced the increase in free xanthophylls in 100 MPa/CUT and 100 MPa/5 min treatments, which were correlated to decreases in xanthophyll ester recoveries in both treatments. On the other hand, recovery of total xanthophyll esters in 100 MPa/CUT and 5 min, 350 MPa/CUT and 5 min and 600 MPa/5 min treated samples was lower (15.9, 6.9, 49.7, 51.9 and 37.6%, respectively) than that in the untreated sample (58.0%), indicating great liberation of xanthophyll esters from the food matrix during the intestinal phase of the in vitro digestion. Hydrocarbon carotene recovery was higher in 350 MPa/5 min treated papaya pulp sample than the other HHP treated samples in different conditions (Table 2). (all-*E*)-β-carotene and (all-*E*)-lycopene showed recovery ranges of 25.1–66.9% and 10.0–71.1%, respectively, in HHP-treated samples. The obtained results are similar to those reported by Cano et al. [21] for pressurized persimmon pulp, where the recoveries of (all-*E*)-β-carotene and (all-*E*)-lycopene were 91 and 63%, respectively. Although there are numerous investigations reporting the recovery and bioaccessibility of carotenoids from different plant material, there is scarce information relating to the effect of non-thermal technologies on the bioaccessibility of individual carotenoids. With the present investigation, we contribute to this research field with relevant information about the effect of HHP technology on the extractability of carotenoids and carotenoid esters from papaya pulp and the study of their bioaccessibility after in vitro gastrointestinal digestion.

### 3.3. Bioccessibility of Carotenoid and Carotenoid Esters in Papaya Submitted to HHP Treatments

Conventionally, thermal processing has been used to reduce microbial load in commercialized food products. However, by applying a heating process, the degradation and isomerization of carotenoids may negatively affect the quality of the processed food. The proposed alternative technologies, such as ultrasounds, high pressure homogenization, pulsed electric field or HHP, have proved their efficiency maintaining food quality attributes while making them safe for consumption [43]. According to the numerous scientific investigations that have been published in the recent years, a relationship between the carotenoid bioaccessibility of food matrices and the effect of non-thermal technologies would be interesting to carry out due to the potential health benefits that it would have in the human body. In the present investigation, the selected papaya variety, Sweet Mary, was studied during the simulated in vitro gastrointestinal digestion, following the INFOGEST^®^ methodology. Table 3 shows the bioaccessibility values of the major individual carotenoids found in the micellar fraction.

Free xanthophylls showed a higher bioaccessibility (%) than the xanthophyll esters and hydrocarbon carotenoids identified in untreated and treated samples. (all-*E*)-violaxanthin, (all-*E*)-zeaxanthin and (all-*E*)-β-cryptoxanthin showed almost identical micellarization efficiency (1.4–3.2%, 1.3–2.6% and 1.4–3.4%, respectively). The non-HHP-treated pulp sample (control) and treated samples showed similar free xanthophylls bioaccessibility (Table 3). The HHP-treated sample at 350 MPa/5 min showed the highest total free xanthophylls incorporated in micelles, with approximately 2.6% of bioaccessibility, while treatment at 100 MPa/5 min produced the lowest free xanthophylls incorporation in micelles, with even less bioaccessibility than the one observed in untreated sample (1.0 and 1.8%, respectively).

(all-*E*)-β-cryptoxanthin laureate was the only xanthophyll ester incorporated into the micellar phase in this step of the digestive process (intestinal phase). The papaya pulp sample treated at 100 MPa/CUT and 100 MPa/5 min did not show any xanthophyll ester incorporation to micelles (micellarization), while, in the rest of the HHP-treated and untreated samples, the (all-*E*)-β-cryptoxanthin laurate bioaccessibility was very low, with a range of 0.1–0.4%.

Finally, among the identified hydrocarbon carotenoids in papaya pulp, (all-*E*)-lycopene was the only carotene incorporated into micelles in all HHP-treated and in the untreated samples; however, this incorporation resulted in very low bioaccessibility percentages (0.1–0.8%). These data agree with several investigations where the reported lycopene bioaccessibility ranges from 0.1–1.6% from different raw vegetables [54]. Other hydrocarbon carotenoids, i.e., (all-*E*)-α-carotene, (all-*E*)-β-carotene and two lycopene isomers, also presented low bioaccessibility (0.1–2.9%), with (all-*E*)-β-carotene being the most bioaccessible hydrocarbon carotenoid (Table 3). (all-*E*)-β-carotene was more bioaccessible than (all-*E*)-α-carotene and the lycopene isomers. In fact, (all-*E*)-α-carotene was only identified in the 350 MPa/CUT treated sample. Treatments at 350 MPa/CUT and 5 min showed to be more effective, favoring the micellarization process, achieving 1.4% and 1.1% of carotenoid bioaccessibility, respectively. The samples submitted to 100 MPa/5 min and 600 MPa/5 min showed the lowest total carotenoid bioaccessibility (0.4–0.6%, respectively). Samples submitted to 100 MPa and 600 MPa at CUT and the untreated sample (control) showed 0.7, 0.8 and 0.7% of carotenoid bioaccessibility, respectively.

The differences in the bioaccessibility of individual carotenoids and carotenoid esters may be associated to HHP treatment. Treatment at 350 MPa during the come-up time (CUT) and 5 min process time resulted to be the most effective to incorporate carotenoids into micelles. However, the chemical structure of each carotenoid represents one of the main factors in the micellarization efficiency, as well as the type of food matrix from which they originate. Several studies have revealed that oxygenated carotenoids (free xanthophylls and xanthophyll esters) are more easily incorporated into the micelles than hydrocarbon carotenoids, because xanthophylls are located on the surface of fat droplets instead of the lipid core, as hydrocarbon carotenoids are [55]. This fact agrees with the results obtained in the present investigation, where we observed higher (all-*E*)-β-cryptoxanthin bioaccessibility compared to (all-*E*)-β-carotene and (all-*E*)-lycopene bioaccessibility, highlighting that carotenoid transfer to the micelles is inversely proportional to the hydrophobicity of the carotenoid [56]. However, Laurora et al. [14] reported the opposite in their investigation, concluding that the form in which β-cryptoxanthin is found in papaya fruits is mainly in esters, which may explain its low bioaccessibility, although higher carotenes bioaccessibility remains unexplained in their investigation. Velderrain-Rodriguez et al. [57] conducted an investigation were they evaluated the effect of dietary fiber in the bioaccessibility of phenolic compounds present in papaya cv. Maradol fruits after a simulated digestion process. They concluded that approximately 40% of the total phenolic compounds was embedded within starchy carbohydrates and 1% of phenolic compounds were associated to dietary fiber. Medium–high pressures may have more influence modifying carbohydrates associated to carotenoids, which might increase their release, facilitating their incorporation into micelles during the intestinal process. Low bioaccessibility has also been reported in lucuma varieties (0.8–0.9%) [52] and in astringent persimmon (0%) [21].

### 3.4. Microstructure of Papaya Fruit and Digesta during In Vitro Digestion

#### 3.4.1. Effect of HHP on Cell Wall and Morphology of Papaya Pulp

**Figure 3:**
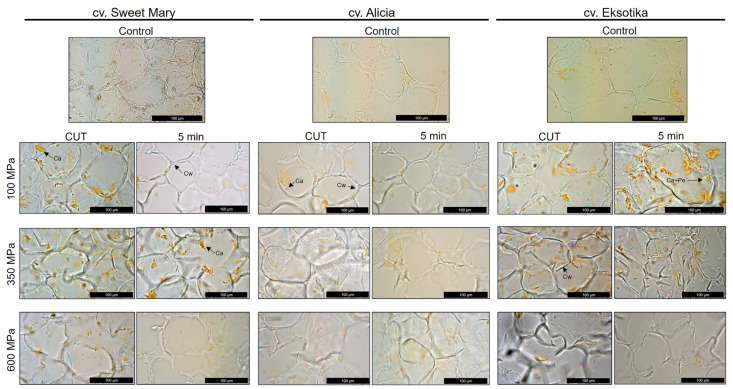
Optical microscopy of Sweet Mary, Alicia and Eksotika papaya (*Carica papaya* L.) varieties of untreated pulps and submitted to HHP treatments of 100, 350 and 600 MPa at CUT and 5 min. Ca, carotenoids; Cw, cell wall; Pe+Ca, carotenoids surrounded by pectin.

Microstructural changes in papaya pulps treated with HHP were analyzed by optical microscopy to assess the effect of this non-thermal treatment on potential carotenoid extractability, stability and bioaccessibility. The parenchyma of the Sweet Mary, Alicia and Eksotika papaya varieties was composed of turgid cells with rounded appearance (Figure 3). The three papaya varieties presented similar high and with cell dimensions in the untreated pulps (Sweet Mary: 98.0 ± 2.4 × 95.3 ± 5.9 μm; Alicia: 90.3 ± 6.4 × 95.0 ± 2.6 μm; Eksotika: 94.6 ± 5.5 × 102.3 ± 6.1 μm). In parenchyma cells, located in the pulp of the fruits, carotenoids could be found inside chromoplasts embedded in the cytoplasm, usually surrounded by pectin due to the high content of soluble dietary fiber in papaya pulp cells [57]. In addition, it has been reported that the deposition of carotenoid within the cell may be different depending on their hydrophobicity degree. Likewise, Schweiggert et al. [58] reported that papaya carotenoids such as β-carotene, β-cryptoxanthin and their esters are disposed in liquid crystalline or lipid-dissolved form inside globular-tubular chromoplasts, differing from lycopene, which is found in solid-crystalline structures deposited into crystalloid chromophores. As a result of these structural differences, they concluded that carotenoids deposited in crystalloid forms presented more difficulties to be liberated than those accumulated in non-crystalloid forms.

The effect of HHP on parenchyma cells significantly affected size, morphology and cell integrity and this was even more accentuated when increasing pressure intensity due to the characteristics of the cell itself, i.e., big cells, large vacuoles, large intercellular spaces and thin cell walls, that make these cells more transformable than those in the collenchyma [59]. At 100 MPa/CUT, a little collapse of the pulp cells occurred, but the intercellular spaces remained similar to the untreated pulps in all papaya varieties. As can be seen in Figure 3 for the sample treated at 100 MPa/5 min, the intercellular spaces disappeared due to cell compression and gas displacement. Cell membranes were still intact but cell wall disruption was starting to occur, an event that was more remarkable at 350 MPa/CUT and even more at 350 MPa/5 min. At this point, the cell membrane was completely ruptured and carotenoids could be observed floating in the intercellular spaces, in a few cases liberated from the pectin; this fact was more noticeable in the Sweet Mary than in the Alicia variety. The carotenoids in the Eksotika variety were also liberated into the intercellular space in high amounts, which may be related to the similar carotenoid content that it showed, compared to the Sweet Mary variety. Under 600 MPa/CUT and 5 min, parenchyma cells were no longer recognizable and carotenoids’ appearance decreased almost to the levels of the control pulps, or even less, at 600 MPa/5 min, especially in the Eksotika variety (Figure 3). This fact could be explained by the degradation and isomerization that carotenoids suffer under such high pressures. On the other hand, pectin traces were still detectable in light microscopy observations, meaning that, although HHP treatments improve carotenoid extractability as reported before [19,21], pectin is still strongly associated with carotenoids, hindering their complete liberation. We intend to further study the effect of HHP on papaya carotenoid extractability by performing complementary treatments to modify the dietary soluble fiber of papaya fruits to present a full insight into carotenoid liberation mechanisms.

#### 3.4.2. Carotenoid Deposition and Factors Affecting Their Stability during In Vitro Digestion

**Figure 4:**
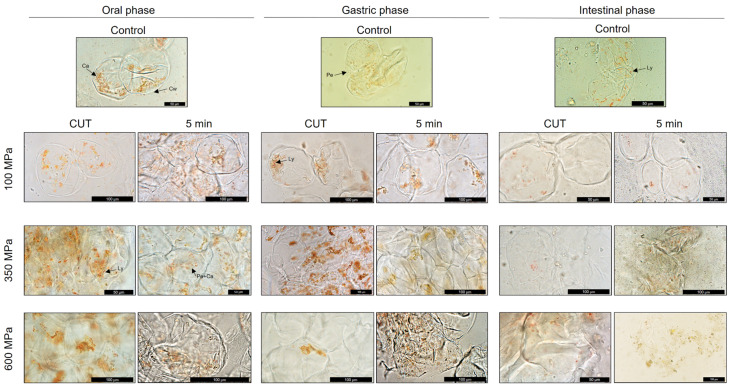
Optical microscopy of Sweet Mary papaya (*Carica papaya* L.) variety of untreated pulps and submitted to HHP treatments of 100, 350 and 600 MPa at CUT and 5 min after in vitro simulated gastrointestinal digestion of digesta fractions. Ca, carotenoids; Cw, cell wall; Ly, lycopene crystalloid formation; Pe, pectin; Pe+Ca, carotenoids surrounded by pectin.

Carotenoids in the digesta fraction were analyzed by light microscopy to evaluate the effects of the non-thermal technology on the distribution and liberation of carotenoids from the food matrix in control (untreated) and HHP-treated papaya (cv. Sweet Mary) pulp during in vitro gastrointestinal digestion (Figure 4). The micellar fractions obtained after each phase of the in vitro digestion were also studied (Figure S5). In Figure 4, it can be seen that, in the oral digesta of the untreated (control) pulp, carotenoids slowly begun to be liberated from the pectin they were embedded in by the action of the α-amylase present in this first phase of the in vitro digestion. At 100 MPa, carotenoids were still deposited in the chromoplasts within the cytoplasm. The cell shape transformed from rounded and turgid to elongated and more pear-shape-like. Under medium pressures (350 MPa), cells totally collapsed, presenting rupture of the cell membrane and, therefore, of the cell wall. Globular and crystalloid carotenoids were found floating into the intercellular space, which was correlated to higher extractability of carotenoids and carotenoid esters after applying medium pressures. In addition, the integrity of lycopene crystalloids was observed and this can be related to the high stability observed at 350 MPa and 600 MPa (Figure 4). This fact can be explained by Schweiggert et al.’s [58] investigation, where they concluded that, during digestion, lycopene crystalloids protect papaya chromoplasts from disruption even under high pressures (600 MPa). We were still able to detect lycopene bioaccesibility at 600 MPa/CUT and 600 MPa/5 min, although, as mentioned above, it was lower than that in the untreated samples.

When analyzing Figure 4 for the sample obtained in the gastric digesta, pectin was very well identified around carotenoids in both untreated and treated papaya pulp at 100 MPa. Actually, higher quantities of pectin substances were identified in this digestion phase due to its capacity of forming hydrogels with the digestion particles in the gastric phase. In addition, in this digestion phase, xanthopyll esters were hydrolyzed, increasing free xanthophylls content, which was more noticeable at 350 MPa. Although no lipids were added during the in vitro digestion process, the formation of small micelles containing carotenoids was observed with light microscopy (Figure S5). Despite the formation of these structures was found only in the micellar fraction of digested papaya pulps treated at 100 MPa/CUT and 350 MPa/CUT, no bioaccessibility differences were detected between treatment and the untreated samples, as it was very low in all of them. It has been demonstrated that micellarization efficiency can be improved with the presence of lipids, even more by selecting a fat source with high content in monounsaturated fatty acids (MUFAs) rather than polyunsaturated fatty acids (PUFAs), in order to form smaller micelles which diffuse more through the intestine, improving carotenoid absorption [14,55]. However, it has to be taken into account that, due to PUFAs hydrophobicity, they might be more related to form micelles with carotenes and xanthophyll esters, while MUFAs might be more likely to integrate free xanthophylls in the micellarization process.

In the intestinal phase, few cell components could be detected due to the activity and interaction of the lipases, bile salts and digestive liquids used in this final phase with the food matrix. Disrupted cell membranes were still observed at 100 MPa, while, at 350 MPa and 600 MPa, just remains of the vegetable cell were identified. Even though globular carotenoids could not be identified in this digestion phase, crystalloid carotenes were detected, explaining the already mentioned lycopene low bioaccessibility.

## 4. Conclusions

This is the first study to report the effects of HHP on individual carotenoid and carotenoid esters present in three new Spanish papaya varieties (cv. Sweet Mary, Alicia and Eksotika) and their stability and bioaccessibility. Papaya pulps were submitted to HHP treatments at low (100 MPa), medium (350 MPa) and high (600 MPa) pressure intensities during CUT and 5 min of time processing. The extractability of free xanthophylls, xanthophyll esters and hydrocarbon carotenoids presented higher values in papaya pulp when subjected to 350 MPa. In the three papaya varieties studied, the HHP treatments produced a significant increase in (all-*E*)-lycopene content (98–1302 μg/100 g fresh weight), followed by the increase in (all-*E*)-β-carotene (51–649 μg/100 g fresh weight), (all-*E*)-β-cyptoxanthin (21–388 μg/100 g fresh weight) and in some β-cyptoxanthin esters, such as β-cyptoxanthin caprate (20–236 μg/100 g fresh weight) and laurate (41–365 μg/100 g fresh weight). Sweet Mary papaya treated pulp showed the highest increase in total carotenoids due to the HHP process (increase in their extractability), compared to the observed carotenoid content in Alicia and Eksotika HHP-treated samples. Among the HHP treatments, the process conducted at 350 MPa during 5 min produced the highest total carotenoid content (4469 ± 124 μg/100 g fresh weight) in cv. Sweet Mary papaya pulp. The results from the in vitro gastrointestinal assay show a high stability of carotenoids and carotenoid esters, but also very low bioaccessibility. Free xanthophllys were more efficientlly micellarizated than xanthophyll esters and hydrocarbon carotenoids due to the polarity of their structures. Different treatments of HHP on papaya pulps influenced cells microstructure, which was correlated to carotenoid and carotenoid ester extractability. Light micrographs showed microstructural changes before and after the in vitro digestion process, highlighting the presence of pectin around the chromoplasts that blocked total carotenoid liberation from the food matrix. In addition, solid crystalloids of lycopene were observed in the intestinal phase of the in vitro digestion samples treated at higher pressure, explaining the low bioaccessibility of the carotene in this process. We intend to perform further studies with papaya fruits by using complementary techniques to improve their low carotenoid bioaccessibility.

## Figures and Tables

**Table 1:** Carotenoid content (μg/100 g fresh weight) and retinol activity equivalents (RAE) of direct pulp extracts of papaya (*Carica papaya* L.) Sweet Mary variety submitted to HHP.

cv. Sweet Mary								
			CUT			5 min		
No	Carotenoid Compound	Control	100 MPa	350 MPa	600 MPa	100 MPa	350 MPa	600 MPa
**1**	(13Z)-violaxanthin	1 ± 0 ^a^	64 ± 4 ^b^	36 ± 2 ^ab^	66 ± 2 ^b^	6 ± 0 ^a^	164 ± 29 ^c^	17 ± 1 ^a^
**2**	(all-*E*)-violaxanthin	3 ± 0 ^a^	85 ± 2 ^d^	65 ± 0 ^c^	50 ± 2 ^b^	15 ± 1 ^a^	122 ± 8 ^e^	13 ± 2 ^a^
**3**	(9Z)-neoxanthin	n.d. ^a^	33 ± 1 ^c^	52 ± 3 ^d^	63 ± 0 ^e^	17 ± 1 ^b^	74 ± 1 ^f^	15 ± 0 ^b^
**4**	(all-*E*)-neoxanthin	n.d. ^a^	23 ± 0 ^b^	n.d. ^a^	18 ± 0 ^b^	14 ± 0 ^b^	87 ± 9 ^c^	n.d. ^a^
**5**	(all-*E*)-lutein	n.d. ^a^	50 ± 3 ^b^	n.d. ^a^	42 ± 3 ^b^	n.d. ^a^	270 ± 11 ^c^	n.d. ^a^
**6**	(all-*E*)-zeaxanthin	n.d. ^a^	33 ± 1 ^b^	n.d. ^a^	97 ± 3 ^c^	28 ± 0 ^b^	225 ± 2 ^d^	n.d. ^a^
**7**	Lutein-5,6-epoxide	n.d. ^a^	87 ± 3 ^c^	n.d. ^a^	47 ± 3 ^b^	n.d. ^a^	143 ± 1 ^d^	n.d. ^a^
**8**	(all-*E*)-antheraxanthin	15 ± 1 ^b^	38 ± 0 ^c^	n.d. ^a^	15 ± 1 ^b^	n.d. ^a^	77 ± 2 ^d^	n.d. ^a^
**9**	(9Z)-violaxanthin	6 ± 0 ^b^	50 ± 3 ^c^	n.d. ^a^	11 ± 1 ^b^	7 ± 1 ^b^	49 ± 2 ^c^	n.d. ^a^
**10**	β-cryptoxanthin-5,6-epoxide	n.d. ^a^	46 ± 1 ^c^	n.d. ^a^	17 ± 1 ^b^	n.d. ^a^	n.d. ^a^	n.d. ^a^
**11**	(9Z)-α-cryptoxanthin	n.d. ^a^	25 ± 1 ^b^	n.d. ^a^	35 ± 0 ^c^	n.d. ^a^	n.d. ^a^	n.d. ^a^
**12**	(all-*E*)-α-cryptoxanthin	3 ± 0 ^c^	4 ± 0 ^c^	n.d. ^a^	6 ± 1 ^d^	2 ± 0 ^b^	n.d. ^a^	n.d. ^a^
**13**	(all-*E*)-β-cryptoxanthin	43 ± 1 ^a^	388 ± 3 ^e^	92 ± 3 ^c^	305 ± 0 ^d^	79 ± 1 ^b^	73 ± 2 ^b^	29 ± 2 ^a^
**14**	α-carotene-5,6-epoxide	3 ± 0 ^b^	24 ± 0 ^c^	n.d. ^a^	n.d. ^a^	21 ± 1 ^c^	40 ± 0 ^d^	n.d. ^a^
**15**	(all-*E*)-luteoxanthin	n.d. ^a^	39 ± 0 ^b^	n.d. ^a^	n.d. ^a^	n.d. ^a^	n.d. ^a^	n.d. ^a^
**16**	(13Z)-α-carotene	14 ± 0 ^c^	14 ± 0 ^c^	n.d. ^a^	22 ± 1 ^f^	19 ± 1 ^d^	77 ± 1 ^e^	5 ± 0 ^b^
**17**	(13Z)-β-carotene	4 ± 0 ^b^	41 ± 0 ^e^	32 ± 0 ^d^	n.d. ^a^	35 ± 1 ^d^	10 ± 1 ^b^	n.d. ^a^
**18**	(all-*E*)-violaxanthin laurate	11 ± 1 ^b^	10 ± 0 ^b^	n.d. ^a^	21 ± 1 ^c^	25 ± 1 ^c^	34 ± 0 ^d^	41 ± 3 ^e^
**19**	α-cryptoxanthin-5,8-epoxide	8 ± 1 ^d^	8 ± 0 ^d^	7 ± 0 ^c^	n.d. ^a^	6 ± 0 ^b^	11 ± 0 ^e^	5 ± 1 ^b^
**20**	(all-*E*)-ζ-carotene	4 ± 0 ^b^	13 ± 0 ^c^	n.d. ^a^	21 ± 1 ^d^	33 ± 2 ^e^	18 ± 1 ^d^	12 ± 1 ^c^
**21**	α-cryptoxanthin-5,8′-epoxide	29 ± 0 ^b^	13 ± 0 ^a^	12 ± 0 ^a^	15 ± 1 ^a^	30 ± 2 ^b^	13 ± 1 ^a^	15 ± 1 ^a^
**22**	(all-*E*)-α-carotene	75 ± 0 ^c^	77 ± 0 ^c^	81 ± 0 ^c^	59 ± 4 ^b^	76 ± 5 ^c^	85 ± 0 ^c^	40 ± 2 ^a^
**23**	(9Z)-α-carotene	3 ± 0 ^b^	n.d. ^a^	n.d. ^a^	n.d. ^a^	n.d. ^a^	8 ± 0 ^c^	n.d. ^a^
**24**	(9Z)-violaxanthin laurate	51 ± 3 ^e^	16 ± 1 ^c^	n.d. ^a^	23 ± 1 ^d^	103 ± 2 ^g^	11 ± 1 ^b^	92 ± 3 ^f^
**25**	(all-*E*)-lutein-3-O-myristate	169 ± 0 ^b^	31 ± 1 ^a^	n.d. ^a^	144 ± 1 ^b^	247 ± 13 ^c^	218 ± 19 ^c^	165 ± 16 ^b^
**26**	(all-*E*)-β-carotene	165 ± 7 ^b^	203 ± 12 ^b^	649 ± 17 ^f^	250 ± 13 ^c^	315 ± 2 ^d^	359 ± 2 ^e^	140 ± 7 ^a^
**27**	(9Z)-β-carotene	6 ± 0 ^a^	n.d. ^a^	49 ± 4 ^d^	20 ± 1 ^b^	n.d. ^a^	33 ± 1 ^c^	7 ± 1 ^a^
**28**	(all-*E*)-violaxanthin dimyristate	36 ± 7 ^b^	n.d. ^a^	69 ± 5 ^d^	46 ± 1 ^c^	n.d. ^a^	54 ± 1 ^c^	48 ± 3 ^c^
**29**	(all-*E*)-antheraxanthin myristate palmitate	43 ± 2 ^b^	33 ± 1 ^a^	126 ± 3 ^f^	69 ± 1 ^c^	27 ± 0 ^a^	94 ± 1 ^e^	81 ± 5 ^d^
**30**	(all-*E*)-violaxanthin palmitate	7 ± 1 ^b^	13 ± 0 ^c^	47 ± 1 ^e^	23 ± 0 ^d^	n.d. ^a^	27 ± 1 ^d^	16 ± 2 ^c^
**31**	(9Z)-neoxanthin dibutyrate	8 ± 0 ^b^	n.d. ^a^	43 ± 1 ^e^	18 ± 0 ^c^	81 ± 2 ^f^	26 ± 0 ^d^	2 ± 2 ^b^
**32**	(all-*E*)-β-cryptoxanthin caprate	82 ± 2 ^b^	32 ± 1 ^a^	236 ± 11 ^f^	111 ± 3 ^c^	135 ± 0 ^d^	170 ± 5 ^e^	72 ± 5 ^b^
**33**	(all-*E)*-violaxanthin myristate palmitate	n.d. ^a^	n.d. ^a^	14 ± 1 ^b^	n.d. ^a^	n.d. ^a^	n.d. ^a^	n.d. ^a^
**34**	(all-*E*)-lutein dimyristate	60 ± 4 ^c^	7 ± 0 ^a^	162 ± 3 ^f^	40 ± 3 ^b^	110 ± 0 ^e^	65 ± 4 ^c^	87 ± 4 ^d^
**35**	(all-*E*)-β-cryptoxanthin laurate	175 ± 10 ^b^	59 ± 1 ^a^	365 ± 13 ^e^	212 ± 2 ^c^	209 ± 0 ^c^	338 ± 9 ^d^	146 ± 9 ^b^
**36**	(all-*E*)-antheraxanthin-3-O palmitate	n.d. ^a^	n.d. ^a^	86 ± 4 ^c^	n.d. ^a^	n.d. ^a^	54 ± 0 ^b^	n.d. ^a^
**37**	(all-*E*)-antheraxanthin laurate myristate	22 ± 2 ^b^	12 ± 0 ^a^	28 ± 1 ^c^	33 ± 2 ^d^	33 ± 1 ^d^	18 ± 1 ^b^	21 ± 2 ^b^
**38**	(all-*E*)-β-cryptoxanthin myristate	19 ± 1 ^b^	5 ± 0 ^a^	62 ± 0 ^f^	25 ± 1 ^d^	22 ± 0 ^c^	42 ± 1 ^e^	16 ± 1 ^b^
**39**	(Z)-lycopene isomer 1	14 ± 0 ^b^	n.d. ^a^	23 ± 1 ^d^	n.d. ^a^	6 ± 0 ^b^	20 ± 1 ^c^	n.d. ^a^
**40**	(all-*E*)-β-cryptoxanthin palmitate	14 ± 0 ^c^	n.d. ^a^	25 ± 2 ^e^	5 ± 1 ^bc^	7 ± 0 ^c^	16 ± 0 ^d^	3 ± 0 ^ab^
**41**	(13Z)-lycopene isomer 2	113 ± 0 ^f^	78 ± 2 ^d^	63 ± 1 ^c^	47 ± 2 ^b^	108 ± 0 ^e^	114 ± 3 ^f^	40 ± 2 ^a^
**42**	(13′Z)-lycopene isomer 3	22 ± 0 ^d^	16 ± 0 ^c^	12 ± 0 ^b^	n.d. ^a^	21 ± 1 ^d^	21 ± 0 ^d^	n.d. ^a^
**43**	(9Z)-lycopene isomer 4	26 ± 0 ^d^	9 ± 0 ^b^	15 ± 0 ^c^	n.d. ^a^	13 ± 0 ^bc^	9 ± 0 ^b^	32 ± 3 ^d^
**44**	(9′Z)-lycopene isomer 5	12 ± 0 ^c^	7 ± 0 ^b^	19 ± 1 ^d^	n.d. ^a^	10 ± 0 ^c^	n.d. ^a^	n.d. ^a^
**45**	(all-*E*)-lycopene	378 ± 5 ^c^	120 ± 4 ^a^	374 ± 15 ^c^	359 ± 15 ^c^	231 ± 4 ^b^	1302 ± 52 ^d^	316 ± 19 ^c^
**46**	(Z)-lycopene isomer 6	23 ± 1 ^b^	n.d. ^a^	n.d. ^a^	n.d. ^a^	n.d. ^a^	n.d. ^a^	n.d. ^a^
**Total free xanthophylls**		109 ± 4 ^a^	985 ± 4 ^d^	264 ± 9 ^b^	787 ± 7 ^c^	204 ± 3 ^b^	1308 ± 66 ^e^	95 ± 6 ^a^
**Total hydrocarbon carotenoids**		696 ± 30 ^a^	602 ± 11 ^a^	1314 ± 37 ^c^	778 ± 29 ^b^	888 ± 1 ^b^	2096 ± 52 ^d^	590 ± 37 ^a^
**Total xanthophyll esters**		859 ± 15 ^b^	219 ± 4 ^a^	1263 ± 43 ^e^	770 ± 3 ^b^	997 ± 18 ^c^	1166 ± 5 ^d^	811 ± 54 ^b^
**Total carotenoids**		1664 ± 48 ^a^	1807 ± 11 ^b^	2841 ± 89 ^d^	2336 ± 33 ^c^	2089 ± 14 ^b^	4469 ± 124 ^e^	1496 ± 99 ^a^
**RAE**		23 ± 1 ^a^	44 ± 1 ^b^	93 ± 3 ^e^	52 ± 1 ^c^	51 ± 0 ^c^	67 ± 1 ^d^	25 ± 2 ^a^

n.d., not detected (detection limit: 0.08 μg/g). Numbers correspond with the HPLC-DAD chromatogram peaks (Figure 1 and Figure S1). Results are expressed as the mean ± standard deviation of duplicate analysis (*n* = 2) of samples from freeze-dried papaya HHP treated pulp. Different superscript letters indicate statistically significant differences of specific content of each compound evaluated (*p* ≤ 0.05), between treatments and the control (untreated) sample. Retinol activity equivalents are calculated according to guidelines of the United States (US) Institute of Medicine [41].

**Table 2:** Carotenoid recovery (%) ^1^ after in vitro simulated gastrointestinal digestion of Sweet Mary papaya (*Carica papaya* L.) variety submitted to HHP treatments (100, 350 and 600 MPa at CUT and 5 min).

Compound	Phase (Digesta)	Non-Treated	100 MPa/CUT	100 MPa/5 min	350 MPa/CUT	350 MPa/5 min	600 MPa/CUT	600 MPa/5 min
**Free xanthophylls**								
(all-*E*)-violaxanthin	Oral	27.4 ± 1.8 ^Ab^	61.7 ± 1.1 ^Af^	65.5 ± 0.1 ^Af^	22.2 ± 0.2 ^Aa^	39.3 ± 0.8 ^Ac^	57.1 ± 0.8 ^Ae^	46.4 ± 0.8 ^Ad^
	Gastric	175.4 ±1.8 ^Bf^	73.0 ± 0.3 ^Bc^	149.1 ± 1.7 ^Ce^	39.6 ± 0.4 ^Ba^	181.9 ± 0.8 ^Cg^	66.4 ± 0.3 ^Bb^	91.6 ± 2.6 ^Cd^
	Intestinal	150.8 ± 3.9 ^Be^	110.9 ± 0.1 ^Cd^	114.8 ± 0.1 ^Bd^	63.4 ± 1.3 ^Ca^	91.0 ± 2.3 ^Bc^	81.5 ± 0.7 ^Cb^	61.0 ± 0.4 ^Ba^
(all-*E*)-zeaxanthin	Oral	15.8 ± 0.9 ^Aa^	33.1 ± 0.4 ^Ab^	39.6 ± 0.7 ^Bc^	51.4 ± 0.2 ^Ce^	43.1 ± 1.0 ^Ad^	51.0 ± 0.4 ^Be^	54.9 ± 0.7 ^Bf^
	Gastric	65.1 ± 3.2 ^Ce^	71.0 ± 0.4 ^Cf^	43.3 ± 0.9 ^Cd^	34.4 ± 0.1 ^Bb^	51.1 ± 0.1 ^Bd^	19.2 ± 0.6 ^Aa^	44.9 ± 0.5 ^Ab^
	Intestinal	47.6 ± 1.6 ^Bb^	59.3 ± 0.2 ^Bd^	22.0 ± 0.1 ^Aa^	22.3 ± 0.3 ^Aa^	93.3 ± 0.5 ^Cf^	89.3± 1.4 ^Ce^	54.1 ± 0.7 ^Bc^
(all-*E*)-antheraxanthin	Oral	210.7± 0.4 ^Cc^	137.6 ± 0.4 ^Ab^	n.d. ^Aa^	n.d. ^Aa^	n.d. ^Aa^	n.d. ^Aa^	138.9 ± 0.8 ^Cb^
	Gastric	75.5 ± 1.7 ^Ab^	147.8 ± 1.2 ^Bd^	n.d. ^Aa^	n.d. ^Aa^	n.d. ^Aa^	n.d. ^Aa^	119.2 ± 1.8 ^Bc^
	Intestinal	129.4 ± 3.6 ^Bc^	262.7± 5.7 ^Cd^	n.d. ^Aa^	n.d. ^Aa^	n.d. ^Aa^	n.d. ^Aa^	76.2 ± 0.5 ^Ab^
(all-*E*)-β-cryptoxanthin	Oral	201.1 ± 0.3 ^Bg^	59.4 ± 1.2 ^Bd^	31.2 ± 0.7 ^Bb^	38.8 ± 0.9 ^Cc^	67.5 ± 0.2 ^Be^	179.0 ± 1.9 ^Cf^	13.7 ± 0.4 ^Ba^
	Gastric	181.0 ± 2.0 ^Ae^	27.7 ± 0.7 ^Ab^	12.5 ± 0.3 ^Aa^	23.3 ± 0.1 ^Ab^	62.5 ± 0.1 ^Ac^	144.9 ± 2.8 ^Bd^	6.9 ± 0.2 ^Aa^
	Intestinal	232.2 ± 2.6 ^Cf^	185.1 ± 4.2 ^Ce^	180.6 ± 1.0 ^Ce^	30.5 ± 0.8 ^Bb^	90.9 ± 0.1 ^Cc^	128.6 ± 0.1 ^Ad^	15.4 ± 0.2 ^Ba^
Total free xanthophylls recovery								
Oral phase		93.5 ± 1.0 ^Ad^	173.0 ± 3.5 ^Ce^	47.4 ± 4.1 ^Ab^	43.2 ± 1.2 ^Cab^	46.7 ± 0.4 ^Ab^	83.0 ± 1.1 ^Bc^	34.3 ± 0.2 ^Ba^
Gastric phase		160.5 ± 8.6 ^Cd^	47.6 ± 0.2 ^Aab^	51.9 ± 1.9 ^Aab^	34.6 ± 0.6 ^Aa^	92.2 ± 1.1 ^Ce^	53.5 ± 0.8 ^Ab^	37.1 ± 0.2 ^Cab^
Intestinal phase		132.8 ± 0.1 ^Bg^	107.0 ± 1.3 ^Bf^	83.9 ± 1.7 ^Be^	37.9 ± 0.1 ^Bb^	72.1 ± 0.7 ^Bc^	77.4 ± 1.2 ^Bd^	27.6 ± 0.4 ^Aa^
**Xanthophyll esters**								
(all-*E*)-lutein-3-O-myristate	Oral	n.d. ^Aa^	69.2 ± 0.7 ^Cd^	n.d. ^Aa^	n.d. ^Aa^	3.6 ± 0.1 ^Ba^	n.d. ^Aa^	75.1 ± 2.4 ^Cc^
	Gastric	n.d. ^Aa^	32.7 ± 0.6 ^Bd^	n.d. ^Aa^	n.d. ^Aa^	2.1 ± 0.0 ^Ab^	n.d. ^Aa^	29.8 ± 0.7 ^Ac^
	Intestinal	n.d. ^Aa^	8.2 ± 0.1 ^Ab^	n.d. ^Aa^	n.d. ^Aa^	41.3 ± 0.3 ^Cc^	n.d. ^Aa^	40.6 ± 0.9 ^Bc^
(9Z)-violaxanthin dimyristate	Oral	41.1 ± 1.0 ^Ab^	95.2 ± 0.2 ^Cf^	n.d. ^Aa^	89.1 ± 0.7 ^Ce^	55.0 ± 0.2 ^Ac^	74.4 ± 1.9 ^Cd^	108.3 ± 2.3 ^Cg^
	Gastric	91.6 ± 5.2 ^Bc^	59.9 ± 1.7 ^Bb^	n.d. ^Aa^	58.2 ± 0.6 ^Ab^	133.5 ± 0.9 ^Bd^	51.5 ± 1.4 ^Ab^	85.6 ± 1.0 ^Bc^
	Intestinal	52.7 ± 1.7 ^Ac^	n.d. ^Aa^	n.d. ^Aa^	69.9 ± 0.3 ^Be^	58.0 ± 0.8 ^Ad^	61.5 ± 1.0 ^Bd^	40.1 ± 1.6 ^Ab^
(all-*E*)-antheraxanthin myristate palmitate	Oral	103.1 ± 1.9 ^Bc^	77.2 ± 5.3 ^Cb^	n.d. ^Aa^	103.9 ± 3.8 ^Cc^	171.4 ± 7.7 ^Cd^	219.5 ± 2.2 ^Ce^	16.6 ± 0.7 ^Ba^
	Gastric	121.7 ± 0.2 ^Cb^	52.5 ± 2.1 ^Bd^	n.d. ^Aa^	77.6 ± 0.5 ^Be^	120.2 ± 0.3 ^Bg^	97.5 ± 0.8 ^Bf^	42.6 ± 1.1 ^Cc^
	Intestinal	94.3 ± 0.8 ^Af^	n.d. ^Aa^	n.d. ^Aa^	40.4 ± 0.2 ^Ac^	80.1 ± 1.0 ^Ae^	47.0 ± 0.4 ^Ad^	11.0 ± 0.0 ^Ab^
(all-*E*)-lutein dimyristate	Oral	10.0 ± 0.2 ^Ab^	86.3 ± 1.7 ^Cf^	0.7 ± 0.0 ^Aa^	39.7 ± 0.1 ^Bd^	19.9 ± 0.6 ^Bc^	72.0 ± 2.6 ^Ae^	192.8 ± 0.3 ^Cg^
	Gastric	18.7 ± 1.9 ^Bb^	51.5 ± 0.3 ^Bd^	7.4 ± 0.0 ^Ba^	29.8 ± 0.9 ^Ac^	12.4 ± 0.7 ^Aa^	96.8 ± 1.9 ^Bf^	91.4 ± 0.7 ^Ae^
	Intestinal	12.2 ± 0.4 ^Ab^	25.5 ± 0.2 ^Ac^	1.4 ± 0.0 ^Aa^	29.8 ± 0.2 ^Ad^	63.3 ± 1.0 ^Ce^	115.5 ± 0.5 ^Cg^	100.9 ± 0.4 ^Bf^
(all-*E*)-β-cryptoxanthin caprate	Oral	75.0 ± 2.5 ^Ac^	59.0± 0.9 ^Cb^	36.8 ± 0.4 ^Ba^	110.9 ± 2.2 ^Cd^	161.0 ± 1.9 ^Cf^	146.4 ± 3.1 ^Ce^	31.5 ± 0.2 ^Ca^
	Gastric	144.9 ± 4.9 ^Ce^	50.8 ± 1.1 ^Bb^	37.4 ± 0.2 ^Ba^	79.1 ± 0.8 ^Bc^	128.6 ± 2.1 ^Bd^	119.7 ± 1.9 ^Bd^	27.9 ± 0.1 ^Ba^
	Intestinal	96.2 ± 1.9 ^Bf^	22.9 ± 0.1 ^Ab^	n.d. ^Aa^	56.3 ± 0.7 ^Ad^	63.1 ± 0.9 ^Ae^	49.1 ± 0.5 ^Ac^	23.9 ± 0.2 ^Ab^
(all-*E*)-β-cryptoxanthin laurate	Oral	37.5 ± 0.0 ^Ab^	65.0 ± 1.3 ^Cd^	38.4 ± 0.1 ^Ab^	56.3 ± 0.5 ^Cc^	112.2 ± 0.3 ^Cf^	104.3 ± 1.1 ^Ce^	33.9 ± 0.0 ^Ca^
	Gastric	96.4 ± 4.5 ^Cd^	45.9± 1.7 ^Bb^	37.5 ± 0.0 ^Ab^	42.0 ± 0.9 ^Bb^	70.0 ± 0.8 ^Bc^	77.5 ± 0.5 ^Bc^	23.7 ± 0.0 ^Aa^
	Intestinal	72.0 ± 0.2 ^Bf^	23.3 ± 0.4 ^Aa^	76.9 ± 0.4 ^Bg^	36.9 ± 0.1 ^Ac^	55.0 ± 0.6 ^Ae^	40.7 ± 0.4 ^Ad^	26.0 ± 0.3 ^Bb^
(all-*E*)-β-cryptoxanthin myristate	Oral	13.5 ± 1.3 ^Aa^	49.1 ± 0.6 ^Cc^	28.4 ± 0.4 ^Ab^	65.1 ± 0.4 ^Be^	102.2 ± 0.6 ^Cf^	129.1 ± 1.6 ^Cg^	54.3 ± 0.1 ^Cd^
	Gastric	48.0 ± 1.6 ^Cb^	42.0 ± 0.6 ^Bb^	84.6 ± 0.5 ^Cd^	94.3 ± 0.4 ^Ce^	72.5 ± 3.0 ^Bc^	68.7 ± 0.5 ^Bc^	25.3 ± 0.1 ^Aa^
	Intestinal	34.9 ± 3.0 ^Bb^	9.9 ± 0.3 ^Aa^	62.2 ± 0.3 ^Bd^	52.2 ± 0.3 ^Ac^	60.8 ± 0.9 ^Ad^	35.0 ± 0.4 ^Ab^	38.6 ± 0.7 ^Bb^
(all-*E*)-β-cryptoxanthin palmitate	Oral	n.d. ^Aa^	101.7 ± 0.7 ^Ce^	66.9 ± 0.4 ^Bb^	94.2 ± 0.0 ^Cd^	78.2 ± 0.6 ^Cc^	102.3 ± 0.9 ^Ce^	n.d. ^Aa^
	Gastric	n.d. ^Aa^	65.5 ± 1.0 ^Bd^	157.5 ± 1.3 ^Ce^	21.2± 0.8 ^Bb^	60.0 ± 0.1 ^Bc^	61.0 ± 0.2 ^Bc^	n.d. ^Aa^
	Intestinal	n.d. ^Aa^	13.0 ± 0.3 ^Ab^	n.d. ^Aa^	12.1 ± 0.3 ^Ab^	n.d. ^Aa^	41.7 ± 0.7 ^Ac^	n.d. ^Aa^
Total xanthophyll ester recovery								
Oral phase		42.3 ± 0.3 ^Ac^	73.0 ± 0.1 ^Cfc^	4.8 ± 0.0 ^Aa^	38.2 ± 0.1 ^Bb^	44.0 ± 0.3 ^Ac^	71.2 ± 0.0 ^Be^	67.4 ± 0.9 ^Cd^
Gastric phase		78.8 ± 1.5 ^Ce^	43.2 ± 0.5 ^Bc^	15.1 ± 0.1 ^Ca^	27.7 ± 0.1 ^Ab^	50.2 ± 1.5 ^Bd^	50.8 ± 0.2 ^Ad^	41.6 ± 0.1 ^Bc^
Intestinal phase		58.0 ± 0.7 ^Be^	15.9 ± 0.2 ^Ab^	6.9 ± 0.0 ^Ba^	49.7 ± 0.1 ^Cd^	51.9 ± 1.0 ^Bd^	69.0 ± 0.5 ^Be^	37.6 ± 0.5 ^Ac^
**Hydrocarbon carotenoids**								
(all-*E*)-α-carotene	Oral	218.5 ± 7.2 ^Be^	43.4 ± 0.5 ^Ab^	43.7 ± 0.4 ^Bb^	49.9 ± 0.8 ^Ab^	86.5 ± 0.1 ^Cc^	189.7 ± 3.0 ^Cd^	23.9 ± 0.0 ^Ca^
	Gastric	245.7± 11.0 ^Bd^	85.2 ± 0.9 ^Bc^	22.5 ± 0.7 ^Aa^	83.6 ± 0.2 ^Cc^	63.8 ± 0.4 ^Bb^	94.6 ± 1.8 ^Bc^	21.1 ± 0.6 ^Ba^
	Intestinal	69.6 ± 1.1 ^Ad^	213.2 ± 1.4 ^Cf^	201.6 ± 1.2 ^Ce^	65.7± 0.7 ^Bd^	25.4 ± 0.1 ^Ab^	56.1 ± 0.7 ^Ac^	13.2 ± 0.4 ^Aa^
(all-*E*)-β-carotene	Oral	50.5 ± 0.5 ^Ac^	57.7± 0.9 ^Cd^	43.6 ± 0.6 ^Bb^	74.4 ± 1.0 ^Ce^	138.4 ± 1.4 ^Cf^	215.7 ± 3.3 ^Cg^	29.6 ± 0.6 ^Ca^
	Gastric	70.0 ± 1.3 ^Bf^	32.5 ± 0.9 ^Ac^	26.1 ± 0.1 ^Ab^	46.4 ± 0.3 ^Bd^	51.1 ± 0.4 ^Ae^	109.5 ± 1.0 ^Bg^	21.1 ± 0.4 ^Aa^
	Intestinal	76.9 ± 0.7 ^Cg^	37.2 ± 0.3 ^Bb^	56.8 ± 0.9 ^Cd^	41.7 ± 0.2 ^Ac^	66.9 ± 0.7 ^Bf^	62.3 ± 1.0 ^Ae^	25.1 ± 0.5 ^Ba^
(13Z)-lycopene isomer 2	Oral	36.3 ± 1.4 ^Aa^	77.2 ± 0.0 ^Bcd^	33.2 ± 0.0 ^Aa^	74.0 ± 2.0 ^Bc^	54.2 ± 0.5 ^Ab^	106.3 ± 2.3 ^Ce^	80.9 ± 0.3 ^Cd^
	Gastric	60.9 ± 0.1 ^Cb^	76.9 ± 0.8 ^Be^	65.2 ± 0.2 ^Cc^	35.5 ± 0.9 ^Aa^	81.2 ± 1.2 ^Bf^	87.0 ± 1.0 ^Bg^	70.3 ± 1.1 ^Bd^
	Intestinal	54.4 ± 1.5 ^Bc^	27.4 ± 0.5 ^Aa^	41.6 ± 0.7 ^Bb^	85.8 ± 0.9 ^Ce^	94.9 ± 0.5 ^Cf^	70.5 ± 0.6 ^Ad^	46.0 ± 1.8 ^Ab^
(9Z)-lycopene isomer 4	Oral	86.9 ± 0.3 ^Ab^	97.8 ± 0.0 ^Bc^	177.3 ± 0.0 ^Bf^	126.6 ± 2.2 ^Ae^	87.5 ± 0.8 ^Ab^	63.1 ± 0.4 ^Aa^	111.6 ± 0.4 ^Ad^
	Gastric	93.0 ± 8.0 ^Aa^	161.2 ± 1.9 ^Cc^	229.2 ± 1.2 ^Cd^	141.6± 1.2 ^Bb^	217.4 ± 2.2 ^Cd^	105.9 ± 1.1 ^Ba^	157.0 ± 1.6 ^Bc^
	Intestinal	89.5 ± 1.3 ^Ac^	57.5 ± 0.3 ^Ab^	41.8 ± 0.9 ^Aa^	171.8 ± 0.6 ^Cf^	144.0 ± 0.4 ^Bd^	151.7 ± 0.1 ^Ce^	236.7 ± 0.1 ^Cg^
(all-*E*)-lycopene	Oral	1.4 ± 0.0 ^Aa^	29.3 ± 0.9 ^Ab^	29.9 ± 0.1 ^Ab^	41.6 ± 0.8 ^Cc^	69.3 ± 0.9 ^Bd^	79.5 ± 0.2 ^Ce^	97.6 ± 1.4 ^Cf^
	Gastric	29.4 ± 0.1 ^Bc^	34.8 ± 0.4 ^Bd^	36.0 ± 0.0 ^Bd^	38.7 ± 0.0 ^Be^	20.1 ± 0.4 ^Aa^	36.8 ± 0.2 ^Bde^	26.8 ± 1.0 ^Ab^
	Intestinal	31.4 ± 0.8 ^Bb^	35.0 ± 0.4 ^Bb^	69.2 ± 1.1 ^Cc^	30.4 ± 0.0 ^Ab^	71.1 ± 2.2 ^Bc^	10.0 ± 0.1 ^Aa^	70.9 ± 0.2 ^Bc^
(Z)-lycopene isomer 6	Oral	68.9 ± 0.5 ^Cc^	176.4 ± 2.5 ^Cf^	76.2 ± 0.3 ^Bd^	114.7 ± 3.4 ^Ce^	42.1 ± 0.3 ^Ab^	n.d. ^Aa^	36.2 ± 0.3 ^Bb^
	Gastric	42.7 ± 0.0 ^Bc^	108.5 ± 0.0 ^Bf^	123.6 ± 0.5 ^Cg^	35.8 ± 0.7 ^Ab^	74.5 ± 0.3 ^Be^	n.d. ^Aa^	56.4 ± 0.1 ^Cd^
	Intestinal	18.8 ± 0.5 ^Ab^	72.0 ± 0.3 ^Ae^	49.8 ± 0.2 ^Ac^	65.4 ± 0.7 ^Bd^	128.1 ± 1.0 ^Cf^	n.d. ^Aa^	n.d. ^Aa^
Total hydrocarbon carotenoid recovery								
Oral phase		34.2 ± 0.2 ^Aa^	39.6 ± 0.1 ^Bb^	33.3 ± 0.1 ^Aa^	46.4 ± 0.7 ^Cc^	72.5 ± 0.2 ^Bd^	89.2 ± 0.3 ^Cf^	83.1 ± 0.7 ^Ce^
Gastric phase		37.3 ± 0.3 ^Bb^	39.6 ± 0.3 ^Bc^	43.3 ± 0.0 ^Bd^	39.4 ± 0.1 ^Bc^	29.8 ± 0.7 ^Aa^	43.9 ± 0.2 ^Bd^	29.0 ± 0.3 ^Aa^
Intestinal phase		37.0 ± 0.0 ^Bc^	34.3 ± 0.0 ^Ab^	68.0 ± 0.3 ^Ce^	36.5 ± 0.0 ^Ac^	71.5 ± 0.4 ^Bf^	17.7 ± 0.2 ^Aa^	60.4 ± 0.2 ^Bd^
Total carotenoids								
Oral phase		23.4 ± 0.1 ^Aa^	49.0 ± 0.1 ^Cc^	22.4 ± 0.2 ^Aa^	44.2 ± 0.5 ^Cb^	64.8 ± 0.0 ^Bd^	84.6 ± 0.1 ^Cf^	76.4 ± 0.3 ^Ce^
Gastric phase		51.1 ± 0.9 ^Ce^	40.5 ± 0.3 ^Bc^	32.5 ± 0.0 ^Ba^	36.2 ± 0.0 ^Ab^	36.5 ± 0.8 ^Ab^	45.6 ± 0.1 ^Bd^	32.8 ± 0.1 ^Aa^
Intestinal phase		45.1 ± 0.1 ^Bd^	32.0 ± 0.1 ^Aa^	44.6 ± 0.0 ^Cc^	39.9 ± 0.0 ^Bb^	66.6 ± 0.0 ^Cf^	31.8 ± 0.1 ^Aa^	52.6 ± 0.1 ^Be^

n.d., not detected. ^1^ Results are expressed as the mean ± standard deviation (*n* = 4). This came from obtaining at least two independent digestions (*n* = 2) and performing the determinations of each two times (*n* = 2). Superscript capital letters indicate statistically significant differences of specific recovery of each compound evaluated (*p* ≤ 0.05), between simulated in vitro digestion phases. Superscript small letters indicate statistically significant differences (*p* ≤ 0.05) between HHP treatments.

**Table 3:** Carotenoid bioaccessibility (%)^1^ after in vitro simulated gastrointestinal digestion of Sweet Mary papaya (*Carica papaya* L.) variety submitted to HHP treatments (100, 350 and 600 MPa at CUT and 5 min).

Compound	Non-Treated	100 MPa/CUT	100 MPa/5 min	350 MPa/CUT	350 MPa/5 min	600 MPa/CUT	600 MPa/5 min
**Free xanthophylls**							
(all-*E*)-violaxanthin	0.6 ± 0.0 ^a^	1.5 ± 0.0 ^b^	1.4 ± 0.0 ^b^	3.2 ± 0.1 ^e^	2.6 ± 0.1 ^d^	2.4 ± 0.1 ^d^	2.0 ± 0.0 ^c^
(all-*E*)-zeaxanthin	1.5 ± 0.0 ^a^	2.6 ± 0.1 ^d^	1.7 ± 0.2 ^ab^	2.1 ± 0.0 ^bc^	2.4 ± 0.1 ^cd^	1.3 ± 0.0 ^a^	1.7 ± 0.0 ^ab^
(all-*E*)-β-cryptoxanthin	3.4 ± 0.1 ^f^	1.9 ± 0.1 ^c^	n.d. ^a^	1.4 ± 0.0 ^b^	2.7 ± 0.1 ^e^	2.4 ± 0.0 ^d^	1.7 ± 0.0 ^c^
**Xanthophyll esters**							
(all-*E*)-β-cryptoxanthin laurate	0.3 ± 0.0 ^d^	n.d. ^a^	n.d. ^a^	0.4 ± 0.0 ^e^	0.3 ± 0.0 ^d^	0.2 ± 0.0 ^c^	0.1 ± 0.0 ^b^
**Hydrocarbon carotenoids**							
(all-*E*)-α-carotene	n.d. ^a^	n.d. ^a^	n.d. ^a^	2.9 ± 0.0 ^b^	n.d. ^a^	n.d. ^a^	n.d. ^a^
(all-*E*)-β-carotene	0.6 ± 0.0 ^c^	n.d. ^a^	n.d. ^a^	n.d. ^a^	0.9 ± 0.0 ^e^	0.8 ± 0.0 ^d^	0.2 ± 0.0 ^b^
(13Z)-lycopene isomer 2	n.d. ^a^	n.d. ^a^	n.d. ^a^	0.9 ± 0.0 ^c^	0.4 ± 0.0 ^b^	n.d. ^a^	n.d. ^a^
(all-*E*)-lycopene	0.3 ± 0.0 ^a^	0.1 ± 0.0 ^a^	0.1 ± 0.0 ^a^	0.5 ± 0.0 ^b^	0.8 ± 0.1 ^c^	0.2 ± 0.0 ^a^	0.1 ± 0.0 ^a^
(Z)-lycopene isomer 6	n.d. ^a^	0.4 ± 0.0 ^c^	0.3 ± 0.0 ^b^	1.6 ± 0.0 ^d^	n.d. ^a^	n.d. ^a^	n.d. ^a^

n.d., not detected. ^1^ Results are expressed as the mean ± standard deviation (*n* = 4). This came from obtaining at least two independent digestions (*n* = 2) and performing the determinations of each two times (*n* = 2). Superscript letters indicate statistically significant differences of specific bioaccessibility of each compound evaluated (*p* ≤ 0.05) between HHP treatments.

## Supplementary Materials

The following are available online at https://www.mdpi.com/article/10.3390/foods10102435/s1, Figure S1: C_30_ reversed-phase chromatograms of carotenoids at 450 nm obtained from Sweet Mary papaya (*Carica papaya* L.) variety of direct (non-saponified) and saponified extracts of (a and b) control, (c and d) 350 MPa/CUT and (e and f) 350 MPa/5 min samples. Peak identities are showed in Table 1. I.S., internal standard, Figure S2: Carotenoid content (μg carotenoids/100 g fresh weight) in direct extracts of (a) cv. Sweet Mary, (b) cv. Alicia and (c) cv. Eksotika papaya (*Carica papaya* L.) pulps submitted to high hydrostatic pressure (HHP: 100, 350 and 600 MPa at CUT and 5 min), Figure S3: Carotenoid content (μg carotenoids/100 g fresh weight) in saponified extracts of (a) cv. Sweet Mary, (b) cv. Alicia and (c) cv. Eksotika papaya (*Carica papaya* L.) pulps submitted to high hydrostatic pressure (HHP: 100, 350 and 600 MPa at CUT and 5 min), Figure S4: C3_0_ reversed-phase chromatograms of carotenoids at 450 nm obtained from cv. Sweet Mary papaya (*Carica papaya* L.) pulp after in vitro gastrointestinal digestion (micellar fraction) of untreated (control) sample—(a) oral phase; (b) gastric phase; (c) intestinal phase—and submitted to high hydrostatic pressure (350 MPa/5min) sample—(d) oral phase; (e) gastric phase; (f) intestinal phase. Peak identities in Table 1, Figure S5: Optical microscopy of Sweet Mary papaya (*Carica papaya* L.) variety of untreated pulps and submitted to 100, 350 and 600 MPa at CUT and 5 min after in vitro simulated gastrointestinal digestion of micellar fractions. Mi, micelle, Table S1: Physical-chemical characteristics in Sweet Mary, Alicia and Eksotika papaya (*Carica papaya* L.) varieties, Table S2: Average of HHP extraction parameters^1^ applied to extract carotenoids from papaya (*Carica papaya* L.) Sweet Mary, Alicia and Eksotika varieties from the Canary Islands (Spain), Table S3: Carotenoid content (μg/100 g fresh weight) ± standard deviation and retinol activity equivalents (RAE) of direct pulp extracts of papaya (*Carica papaya* L.) Alicia variety submitted to HHP, Table S4: Carotenoid content (μg/100 g fresh weight) ± standard deviation and retinol activity equivalents (RAE) of direct pulp extracts of papaya (*Carica papaya* L.) Eksotika variety submitted to HHP, Table S5: Carotenoid content (μg/100 g fresh weight) ± standard deviation of papaya (*Carica papaya* L.) pulp cv. Sweet Mary submitted to HHP treatments (100, 350 and 600 MPa at CUT and 5 min) after each phase of in vitro simulated gastrointestinal digestion.
