# Supplementary material for: Effect of High Hydrostatic Pressure on the Extractability and Bioaccessibility of Carotenoids and Their Esters from Papaya (Carica papaya L.) and Its Impact on Tissue Microstructure
Source: Foods. 2021 Oct 13;10(10):2435. doi: 10.3390/foods10102435 (PMC8535580; doi:10.3390/foods10102435)
Supplement: Supplementary file 1 [file foods-10-02435-s001.zip › Supplementary Table S1 (3).pdf]

Table S1. Physical-chemical characteristics in Sweet Mary, Alicia and Eksotika papaya (*Carica papaya* L.) varieties.

**Characteristic**[1]	**cv. Sweet Mary**	**cv. Alicia**	**cv. Eksotika**
Total weight of whole fruit (g)	1124.0 ± 214.2[a]	1286.1 ± 155.3[a]	1148.2 ± 204.3[a]
Apical calibre (cm)	19.1 ± 5.2[a]	20.5 ± 0.8[a]	19.4 ± 2.0[a]
Equatorial calibre (cm)	10.7 ± 0.7[a]	11.6 ± 0.3[a]	11.5 ± 0.4[a]
Titratable acidity[2]	0.184 ± 0.000[a]	0.182 ± 0.005[a]	0.190 ± 0.002[a]
pH	5.275 ± 0.007[a]	5.350 ± 0.028[b]	5.430 ± 0.000[c]
Soluble solids (°Brix at 20 °C)	12.2 ± 0.2[b]	11.2 ± 0.1[a]	12.1 ± 0.2[b]
Moisture content (% wet basis)	83.4 ± 0.2[a]	84.6 ± 0.3[b]	85.1 ± 0.2[c]
Pulp color parameters			
L^*	63.1 ± 1.1[a]	62.3 ± 0.9[a]	63.0 ± 3.7[a]
a^*	28.1 ± 1.2[c]	23.9 ± 0.3[b]	19.8 ± 1.0[a]
b^*	42.8 ± 2.1[b]	40.4 ± 1.1[b]	30.8 ± 0.8[a]
hue angle (h^*)	56.6 ± 0.3[b]	59.3 ± 1.0[c]	57.3 ± 0.7[b]
Peel color parameters			
L^*	56.0 ± 0.7[a]	50.3 ± 1.1[a]	59.0 ± 2.2[a]
a^*	13.7 ± 0.9[a]	15.2 ± 0.5[a]	14.1 ± 0.5[a]
b^*	24.4 ± 0.6[a]	22.9 ± 1.4[a]	26.4 ± 3.6[a]
hue angle (h^*)	60.8 ± 1.1[b]	56.4 ± 1.3[a]	61.7 ± 2.5[b]

[1] Values are the mean of three independent determinations ± standard deviation. Superscript letter indicates statistically significant differences ($p \leq 0.05$).

[2] g citric acid/100 g fresh weight.
