# Supplementary material for: Effect of High Hydrostatic Pressure on the Extractability and Bioaccessibility of Carotenoids and Their Esters from Papaya (Carica papaya L.) and Its Impact on Tissue Microstructure
Source: Foods. 2021 Oct 13;10(10):2435. doi: 10.3390/foods10102435 (PMC8535580; doi:10.3390/foods10102435)
Supplement: Supplementary file 1 [file foods-10-02435-s001.zip › Supplementary Table S2 (3).pdf]

Table S2. Average of HHP extraction parameters[1] applied to extract carotenoids from papaya (*Carica papaya* L.) Sweet Mary, Alicia and Eksotika varieties from the Canary Islands (Spain).

Target hold pressure (MPa)	Target hold time (min)	Target extraction temperature (°C)	AUC pressure (MPa x min)	AUC temperature (°C x min)	Δt (min)	Mean effective influence of pressure $P_{extr.}$ (MPa)	Mean effective influence of temperature $T_{extr.}$ (°C)	Mean pressure at hold time (MPa)	Mean temperature at hold time (°C)	Δt at hold time (min)
100	1 s	25	40.4	23.1	1.0	40.0	23.0	101.5 ± 4.4	24.5 ± 0.3	0.1
	5	25	530.8	149.8	5.9	90.0	25.4	99.4 ± 2.7	25.4 ± 0.6	5.1
350	1 s	25	390.0	68.6	2.6	150.0	26.4	340.8 ± 5.6	30.6 ± 0.2	0.3
	5	25	2038.0	197.3	7.2	282.4	27.3	348.6 ± 2.1	28.1 ± 1.3	5.0
600	1 s	25	903.7	93.8	3.5	259.4	26.9	576.9 ± 9.2	32.0 ± 1.3	0.4
	5	25	3749.9	235.4	8.4	448.2	28.1	592.6 ± 9.9	29.4 ± 2.0	5.2

[1] Target hold pressure and time-extraction pressure and time that was anticipated at hold time and set by command console of the high pressure unit.

Target extraction temperature-anticipated temperature of entire extraction during entire length of extraction time for one batch of samples.

AUC pressure-the size of area under the pressure curve for one batch of samples during entire length of extraction.

AUC temperature-the size of area under the temperature curve for one batch of samples during entire length of extraction.

Δt-entire length of extraction time for one batch of samples.

Mean effective influence of pressure $P_{extr.}$ - influence of pressure for entire extraction obtain by dividing the AUC pressure by Δt.

Mean effective influence of temperature $T_{extr.}$ - influence of temperature for entire extraction obtain by dividing the AUC temperature by Δt.

Mean pressure at hold time – average value of pressure measured by probe within HPE cylinder during the hold time.

Mean temperature at hold time – average value of temperature measured by probe within HPE cylinder during the hold time.

Δt at hold time – length of hold time during which oscillation in target extraction pressure were less than 5% from what was inputted in the command console. The above parameters were calculated following the indications reported by Putnik et al. [61].
