# Supplementary material for: Effect of High Hydrostatic Pressure on the Extractability and Bioaccessibility of Carotenoids and Their Esters from Papaya (Carica papaya L.) and Its Impact on Tissue Microstructure
Source: Foods. 2021 Oct 13;10(10):2435. doi: 10.3390/foods10102435 (PMC8535580; doi:10.3390/foods10102435)
Supplement: Supplementary file 1 [file foods-10-02435-s001.zip › Supplementary Table S3 (3).pdf]

Table S3. Carotenoid content (μg/100 g fresh weight) ± standard deviation and retinol activity equivalents (RAE) of direct pulp extracts of papaya (*Carica papaya* L.) Alicia variety submitted to HHP.

		**cv. Alicia**						
			**CUT (come-up time)**			**5 min**		
**No**	Carotenoid compound	**Control**	**100 MPa**	**350 MPa**	**600 MPa**	**100 MPa**	**350 MPa**	**600 MPa**
**1**	(13Z)-violaxanthin	n.d.[a]	54 ± 1[c]	271 ± 8[f]	35 ± 1[b]	61 ± 1[c]	215 ± 2[e]	87 ± 2[d]
**2**	(all-*E*)-violaxanthin	n.d.[a]	n.d.[a]	264 ± 2[f]	32 ± 1[b]	77 ± 1[e]	49 ± 1[c]	69 ± 2[d]
**3**	(9Z)-neoxanthin	n.d.[a]	n.d.[a]	13 ± 0[b]	13 ± 1[b]	42 ± 2[c]	12 ± 1[b]	n.d.[a]
**4**	(all-*E*)-neoxanthin	n.d.[a]	n.d.[a]	n.d.[a]	11 ± 0[b]	n.d.[a]	n.d.[a]	40 ± 1[c]
**5**	(all-*E*)-lutein	n.d.[a]	n.d.[a]	n.d.[a]	19 ± 0[b]	n.d.[a]	n.d.[a]	44 ± 1[c]
**6**	(all-*E*)-zeaxanthin	15 ± 1[b]	115 ± 3[e]	n.d.[a]	12 ± 0[b]	24 ± 1[c]	50 ± 4[d]	n.d.[a]
**7**	Lutein-5,6-epoxide	n.d.[a]	n.d.[a]	n.d.[a]	n.d.[a]	n.d.[a]	n.d.[a]	n.d.[a]
**8**	(all-*E*)-antheraxanthin	n.d.[a]	n.d.[a]	n.d.[a]	n.d.[a]	9 ± 0[b]	n.d.[a]	n.d.[a]
**9**	(9Z)-violaxanthin	n.d.[a]	n.d.[a]	n.d.[a]	n.d.[a]	12 ± 1[b]	12 ± 1[b]	n.d.[a]
**10**	β-cryptoxanthin-5, 6-epoxide	n.d.[a]	n.d.[a]	n.d.[a]	n.d.[a]	n.d.[a]	8 ± 1[b]	n.d.[a]
**11**	(9Z)-α-cryptoxanthin	n.d.[a]	n.d.[a]	n.d.[a]	n.d.[a]	42 ± 0[c]	6 ± 0[b]	n.d.[a]
**12**	(all-*E*)-α-cryptoxanthin	5 ± 0[b]	n.d.[a]	n.d.[a]	n.d.[a]	55 ± 0[c]	n.d.[a]	n.d.[a]
**13**	(all-*E*)-β-cryptoxanthin	43 ± 3[b]	47 ± 2[b]	21 ± 1[a]	52 ± 1[b]	90 ± 3[d]	63 ± 4[c]	51 ± 2[b]
**14**	α-carotene-5,6-epoxide	6 ± 0[b]	24 ± 1[c]	13 ± 0[b]	25 ± 1[c]	21 ± 1[c]	n.d.[a]	46 ± 0[d]
**15**	(all-*E*)-luteoxanthin	n.d.[a]	n.d.[a]	n.d.[a]	n.d.[a]	98 ± 0[b]	n.d.[a]	n.d.[a]
**16**	(13Z)-α-carotene	14 ± 0[c]	n.d.[a]	6 ± 0[b]	10 ± 0[c]	48 ± 2[d]	10 ± 0[c]	95 ± 0[e]
**17**	(13Z)-β-carotene	5 ± 0[ab]	n.d.[a]	4 ± 0[ab]	28 ± 1[c]	79 ± 3[e]	9 ± 0[b]	73 ± 1[d]
**18**	(all-*E*)-violaxanthin laurate	8 ± 2[ab]	n.d.[a]	16 ± 0[b]	27 ± 0[c]	115 ± 2[e]	18 ± 1[b]	59 ± 2[d]
**19**	α-cryptoxanthin-5, 8-epoxide	3 ± 0[b]	n.d.[a]	n.d.[a]	n.d.[a]	32 ± 2[c]	5 ± 0[b]	n.d.[a]
**20**	(all-*E*)-ζ-carotene	n.d.[a]	n.d.[a]	8 ± 2[b]	n.d.[a]	n.d.[a]	n.d.[a]	n.d.[a]
**21**	α-cryptoxanthin-5, 8′-epoxide	13 ± 2[c]	33 ± 2[d]	7 ± 0[b]	n.d.[a]	11 ± 0[c]	n.d.[a]	n.d.[a]
**22**	(all-*E*)-α-carotene	75 ± 1[c]	31 ± 2[b]	26 ± 2[a]	19 ± 1[a]	33 ± 0[b]	19 ± 1[a]	105 ± 0[d]
**23**	(9Z)-α-carotene	n.d.[a]	n.d.[a]	16 ± 0[b]	n.d.[a]	n.d.[a]	n.d.[a]	35 ± 0[c]
**24**	(9Z)-violaxanthin laurate	n.d.[a]	44 ± 3[d]	103 ± 1[f]	22 ± 0[c]	11 ± 1[b]	14 ± 0[b]	60 ± 0[e]
**25**	(all-*E*)-lutein-3-O-myristate	211 ± 1[f]	124 ± 0[d]	32 ± 0[a]	86 ± 3[c]	62 ± 2[b]	60 ± 0[b]	139 ± 1[e]
**26**	(all-*E*)-β-carotene	120 ± 2[d]	120 ± 5[d]	76 ± 1[b]	95 ± 6[c]	69 ± 1[b]	53 ± 1[a]	77 ± 0[b]
**27**	(9Z)-β-carotene	5 ± 0[b]	17 ± 0[d]	n.d.[a]	26 ± 0[e]	12 ± 1[c]	n.d.[a]	5 ± 0[b]

**28**	(all-*E*)-violaxanthin dimyristate	41 ± 2^{c}	51 ± 1^{d}	n.d.^{a}	47 ± 1^{c}	12 ± 0^{b}	15 ± 1^{b}	15 ± 0^{b}
**29**	(all-*E*)-antheraxanthin myristat palmitate	43 ± 0^{d}	69 ± 2^{e}	42 ± 1^{d}	35 ± 1^{c}	17 ± 1^{b}	19 ± 1^{b}	7 ± 1^{a}
**30**	(all-*E*)-violaxanthin palmitate	11 ± 1^{d}	50 ± 0^{f}	12 ± 1^{d}	9 ± 0^{c}	n.d.^{a}	18 ± 0^{e}	6 ± 0^{b}
**31**	(9Z)- neoxanthin dibutyrate	16 ± 0^{a}	24 ± 1^{c}	36 ± 0^{d}	12 ± 0^{a}	20 ± 1^{b}	14 ± 1^{a}	37 ± 0^{d}
**32**	(all-*E*)-β-cryptoxanthin caprate	70 ± 2^{d}	90 ± 2^{e}	36 ± 2^{c}	61 ± 1^{d}	23 ± 1^{ab}	20 ± 0^{a}	27 ± 2^{b}
**33**	(all-*E*)-violaxanthin myristate palmitate	10 ± 0^{b}	n.d.^{a}	n.d.^{a}	15 ± 0^{b}	n.d.^{a}	62 ± 2^{c}	n.d.^{a}
**34**	(all-*E*)-lutein dimyristate	62 ± 4^{d}	91 ± 5^{e}	17 ± 0^{bc}	23 ± 1^{c}	n.d.^{a}	12 ± 0^{b}	12 ± 0^{b}
**35**	(all-*E*)-β-cryptoxanthin laurate	168 ± 4^{d}	177 ± 0^{d}	106 ± 1^{b}	144 ± 4^{c}	48 ± 1^{a}	49 ± 3^{a}	44 ± 0^{a}
**36**	(all-*E*)-antheraxanthin-3-O palmitate	n.d.^{a}	n.d.^{a}	n.d.^{a}	34 ± 3^{b}	n.d.^{a}	n.d.^{a}	n.d.^{a}
**37**	(all-*E*)-antheraxanthin laurate myristate	17 ± 2^{b}	27 ± 1^{c}	82 ± 1^{d}	28 ± 1^{c}	n.d.^{a}	18 ± 1^{b}	20 ± 0^{b}
**38**	(all-*E*)-β-cryptoxanthin myrista	13 ± 0^{c}	24 ± 2^{d}	12 ± 0^{c}	15 ± 0^{c}	n.d.^{a}	8 ± 1^{b}	6 ± 0^{b}
**39**	(Z)-lycopene isomer 1	12 ± 0^{b}	16 ± 1^{b}	119 ± 2^{d}	38 ± 3^{c}	n.d.^{a}	n.d.^{a}	n.d.^{a}
**40**	(all-*E*)-β-cryptoxanthin palmita	5 ± 0^{b}	5 ± 1^{b}	8 ± 0^{c}	6 ± 0^{b}	n.d.^{a}	n.d.^{a}	n.d.^{a}
**41**	(13Z)-lycopene isomer 2	194 ± 3^{e}	68 ± 2^{c}	120 ± 4^{d}	192 ± 4^{e}	35 ± 2^{b}	73 ± 1^{c}	19 ± 1^{a}
**42**	(13′Z)-lycopene isomer 3	25 ± 0^{c}	n.d.^{a}	n.d.^{a}	17 ± 1^{b}	n.d.^{a}	n.d.^{a}	n.d.^{a}
**43**	(9Z)-lycopene isomer 4	21 ± 2^{b}	15 ± 0^{b}	128 ± 2^{c}	172 ± 5^{d}	n.d.^{a}	21 ± 2^{b}	17 ± 1^{b}
**44**	(9′Z)-lycopene isomer 5	21 ± 1^{b}	20 ± 1^{b}	n.d.^{a}	8 ± 0^{a}	n.d.^{a}	173 ± 7^{c}	n.d.^{a}
**45**	(all-*E*)-lycopene	328 ± 2^{bc}	279 ± 13^{b}	367 ± 4^{c}	437 ± 0^{e}	410 ± 4^{d}	257 ± 13^{b}	211 ± 3^{a}
**46**	(Z)-lycopene isomer 6	16 ± 0^{b}	n.d.^{a}	217 ± 2^{c}	n.d.^{a}	n.d.^{a}	n.d.^{a}	n.d.^{a}
**Total free xanthophylls**		85 ± 6^{a}	249 ± 8^{c}	577 ± 6^{g}	174 ± 2^{b}	552 ± 4^{f}	420 ± 3^{e}	291 ± 6^{d}
**Total hydrocarbon carotenoids**		674 ± 20^{b}	589 ± 24^{a}	1101 ± 14^{c}	1068 ± 21^{c}	708 ± 13^{b}	614 ± 19^{a}	684 ± 5^{b}
**Total xanthophyll esters**		836 ± 14^{e}	775 ± 12^{e}	502 ± 3^{c}	564 ± 5^{d}	308 ± 4^{a}	326 ± 8^{a}	431 ± 3^{b}
**Total carotenoids**		1595 ± 40^{b}	1613 ± 44^{b}	2179 ± 6^{d}	1806 ± 29^{c}	1567 ± 20^{b}	1360 ± 30^{a}	1406 ± 2^{a}
**RAE**		18 ± 1^{b}	27 ± 1^{e}	17 ± 0^{b}	24 ± 1^{d}	21 ± 1^{c}	12 ± 1^{a}	27 ± 0^{e}

n.d. not detected (detection limit: 0.08 μg/g). Numbers correspond with the HPLC-DAD chromatogram peaks (Figure 1 and Figure S1). Results are expressed as the mean ± standard deviation of duplicate analysis (n = 2) of samples from freeze-dried papaya HHP treated pulp. Different superscript letters indicate statistically significant differences of specific content of each compound evaluated ($p \leqslant 0.05$), between treatments and the control (untreated) sample. Retinol activity equivalents are calculated according to guidelines of the United States (US) Institute of Medicine [41].
