# Supplementary material for: Effect of High Hydrostatic Pressure on the Extractability and Bioaccessibility of Carotenoids and Their Esters from Papaya (Carica papaya L.) and Its Impact on Tissue Microstructure
Source: Foods. 2021 Oct 13;10(10):2435. doi: 10.3390/foods10102435 (PMC8535580; doi:10.3390/foods10102435)
Supplement: Supplementary file 1 [file foods-10-02435-s001.zip › Supplementary Table S4 (2).pdf]

Table S4. Carotenoid content (µg/100 g fresh weight) ± standard deviation and retinol activity equivalents (RAE) of direct pulp extracts of papaya (*Carica papaya* L.) Eksotika variety submitted to HHP.

			**cv. Eksotika**					
			**CUT (come-up time)**			**5 min**		
**No**	Carotenoid compound	**Control**	**100 MPa**	**350 MPa**	**600 MPa**	**100 MPa**	**350 MPa**	**600 MPa**
**1**	(13Z)-violaxanthin	n.d.^{a}	617 ± 16^{f}	186 ± 1^{d}	66 ± 2^{b}	110 ± 1^{c}	225 ± 0^{e}	22 ± 0^{a}
**2**	(all-*E*)-violaxanthin	n.d.^{a}	114 ± 7^{e}	120 ± 2^{e}	76 ± 1^{d}	51 ± 1^{b}	67 ± 1^{c}	38 ± 0^{a}
**3**	(9Z)-neoxanthin	n.d.^{a}	n.d.^{a}	n.d.^{a}	n.d.^{a}	64 ± 0^{c}	n.d.^{a}	11 ± 0^{b}
**4**	(all-*E*)-neoxanthin	n.d.^{a}	n.d.^{a}	n.d.^{a}	n.d.^{a}	11 ± 1^{b}	36 ± 0^{c}	n.d.^{a}
**5**	(all-*E*)-lutein	n.d.^{a}	56 ± 0^{c}	n.d.^{a}	n.d.^{a}	41 ± 2^{b}	n.d.^{a}	n.d.^{a}
**6**	(all-*E*)-zeaxanthin	28 ± 2^{b}	n.d.^{a}	n.d.^{a}	n.d.^{a}	n.d.^{a}	68 ± 2^{c}	31 ± 1^{b}
**7**	Lutein-5,6-epoxide	n.d.^{a}	n.d.^{a}	n.d.^{a}	n.d.^{a}	n.d.^{a}	n.d.^{a}	n.d.^{a}
**8**	(all-*E*)-antheraxanthin	n.d.^{a}	n.d.^{a}	n.d.^{a}	n.d.^{a}	n.d.^{a}	15 ± 0^{b}	n.d.^{a}
**9**	(9Z)-violaxanthin	n.d.^{a}	n.d.^{a}	n.d.^{a}	n.d.^{a}	9 ± 1^{b}	n.d.^{a}	n.d.^{a}
**10**	β-cryptoxanthin-5, 6-epoxide	n.d.^{a}	n.d.^{a}	n.d.^{a}	n.d.^{a}	n.d.^{a}	n.d.^{a}	n.d.^{a}
**11**	(9Z)-*α*-cryptoxanthin	n.d.^{a}	n.d.^{a}	n.d.^{a}	n.d.^{a}	n.d.^{a}	n.d.^{a}	n.d.^{a}
**12**	(all-*E*)-*α*-cryptoxanthin	7 ± 0^{b}	n.d.^{a}	n.d.^{a}	n.d.^{a}	5 ± 0^{b}	n.d.^{a}	n.d.^{a}
**13**	(all-*E*)-β-cryptoxanthin	41 ± 1^{ab}	119 ± 1^{e}	27 ± 2^{a}	51 ± 0^{b}	81 ± 0^{d}	77 ± 0^{c}	25 ± 1^{a}
**14**	*α*-carotene-5,6-epoxide	9 ± 1^{b}	12 ± 1^{c}	10 ± 0^{b}	n.d.^{a}	n.d.^{a}	17 ± 0^{d}	10 ± 0^{b}
**15**	(all-*E*)-luteoxanthin	n.d.^{a}	n.d.^{a}	n.d.^{a}	n.d.^{a}	n.d.^{a}	n.d.^{a}	n.d.^{a}
**16**	(13Z)-*α*-carotene	14 ± 1^{b}	56 ± 1^{e}	21 ± 0^{c}	n.d.^{a}	33 ± 0^{b}	38 ± 0^{d}	14 ± 1^{b}
**17**	(13Z)-β-carotene	5 ± 0^{b}	n.d.^{a}	8 ± 0^{b}	n.d.^{a}	8 ± 0^{b}	14 ± 1^{c}	8 ± 0^{b}
**18**	(all-*E*)-violaxanthin laurate	20 ± 2^{b}	n.d.^{a}	34 ± 1^{d}	n.d.^{a}	31 ± 1^{c}	39 ± 0^{e}	17 ± 0^{b}
**19**	*α*-cryptoxanthin-5, 8-epoxide	14 ± 1^{c}	n.d.^{a}	n.d.^{a}	n.d.^{a}	16 ± 1^{c}	31 ± 1^{d}	9 ± 0^{b}
**20**	(all-*E*)-ζ-carotene	56 ± 1^{d}	n.d.^{a}	n.d.^{a}	n.d.^{a}	14 ± 0^{b}	27 ± 1^{c}	15 ± 0^{b}
**21**	*α*-cryptoxanthin-5, 8′-epoxide	23 ± 1^{b}	32 ± 1^{c}	25 ± 2^{b}	18 ± 0^{a}	26 ± 1^{b}	38 ± 1^{d}	18 ± 1^{a}
**22**	(all-*E*)-*α*-carotene	79 ± 1^{d}	28 ± 0^{a}	51 ± 1^{b}	70 ± 1^{c}	80 ± 1^{d}	86 ± 0^{e}	50 ± 0^{b}
**23**	(9Z)-*α*-carotene	4 ± 0^{ab}	45 ± 1^{c}	15 ± 1^{b}	n.d.^{a}	n.d.^{a}	15 ± 1^{b}	n.d.^{a}
**24**	(9Z)-violaxanthin laurate	59 ± 1^{f}	10 ± 1^{a}	26 ± 0^{c}	29 ± 0^{d}	20 ± 1^{b}	39 ± 0^{e}	40 ± 0^{e}
**25**	(all-*E*)-lutein-3-O-myristate	262 ± 5^{g}	77 ± 1^{a}	102 ± 0^{b}	161 ± 1^{d}	198 ± 10^{e}	222 ± 6^{f}	125 ± 2^{c}
**26**	(all-*E*)-β-carotene	170 ± 9^{d}	85 ± 1^{b}	148 ± 0^{c}	145 ± 2^{c}	242 ± 2^{e}	151 ± 5^{c}	56 ± 1^{a}
**27**	(9Z)-β-carotene	n.d.^{a}	42 ± 0^{b}	44 ± 0^{c}	58 ± 0^{d}	76 ± 0^{e}	40 ± 1^{b}	20 ± 1^{a}

**28**	(all-*E*)-violaxanthin dimyrista	48 ± 3^{e}	n.d.^{a}	39 ± 2^{d}	34 ± 1^{c}	30 ± 1^{b}	43 ± 1^{e}	34 ± 0^{c}
**29**	(all-*E*)-antheraxanthin myrista palmitate	53 ± 2^{c}	9 ± 0^{a}	59 ± 0^{d}	78 ± 0^{e}	102 ± 2^{f}	54 ± 1^{c}	27 ± 1^{b}
**30**	(all-*E*)-violaxanthin palmitate	20 ± 1^{e}	n.d.^{a}	20 ± 0^{e}	16 ± 1^{d}	13 ± 1^{c}	15 ± 1^{cd}	10 ± 0^{b}
**31**	(9Z)- neoxanthin dibutyrate	28 ± 2^{d}	n.d.^{a}	26 ± 0^{d}	32 ± 1^{e}	20 ± 0^{c}	8 ± 0^{b}	9 ± 0^{b}
**32**	(all-*E*)-β-cryptoxanthin caprat	90 ± 2^{d}	24 ± 0^{a}	103 ± 0^{e}	90 ± 1^{d}	113 ± 1^{f}	81 ± 0^{c}	40 ± 0^{b}
**33**	(all-*E*)-violaxanthin myristate palmitate	12 ± 0^{b}	n.d.^{a}	n.d.^{a}	n.d.^{a}	n.d.^{a}	n.d.^{a}	n.d.^{a}
**34**	(all-*E*)-lutein dimyristate	80 ± 2^{g}	n.d.^{a}	56 ± 0^{f}	49 ± 1^{d}	52 ± 1^{e}	38 ± 1^{c}	29 ± 0^{b}
**35**	(all-*E*)-β-cryptoxanthin laurat	223 ± 8^{e}	41 ± 0^{a}	215 ± 4^{e}	201 ± 3^{d}	249 ± 7^{f}	156 ± 0^{c}	102 ± 2^{b}
**36**	(all-*E*)-antheraxanthin-3-O palmitate	n.d.^{a}	n.d.^{a}	n.d.^{a}	n.d.^{a}	n.d.^{a}	n.d.^{a}	n.d.^{a}
**37**	(all-*E*)-antheraxanthin laurate myristate	17 ± 1^{b}	n.d.^{a}	62 ± 0^{e}	25 ± 1^{c}	31 ± 2^{d}	32 ± 0^{d}	17 ± 0^{b}
**38**	(all-*E*)-β-cryptoxanthin myrist	21 ± 1^{d}	n.d.^{a}	30 ± 0^{f}	25 ± 0^{e}	23 ± 1^{d}	9 ± 1^{b}	12 ± 0^{c}
**39**	(Z)-lycopene isomer 1	23 ± 2^{c}	47 ± 2^{d}	55 ± 1^{e}	n.d.^{a}	12 ± 1^{b}	n.d.^{a}	n.d.^{a}
**40**	(all-*E*)-β-cryptoxanthin palmit	10 ± 0^{c}	n.d.^{a}	16 ± 0^{c}	n.d.^{a}	5 ± 0^{b}	n.d.^{a}	n.d.^{a}
**41**	(13Z)-lycopene isomer 2	198 ± 6^{f}	n.d.^{a}	211 ± 3^{f}	87 ± 1^{d}	75 ± 2^{c}	104 ± 7^{e}	30 ± 0^{b}
**42**	(13´Z)-lycopene isomer 3	29 ± 1^{c}	47 ± 2^{d}	16 ± 0^{b}	n.d.^{a}	16 ± 0^{b}	n.d.^{a}	n.d.^{a}
**43**	(9Z)-lycopene isomer 4	30 ± 2^{b}	119 ± 2^{e}	86 ± 0^{d}	n.d.^{a}	n.d.^{a}	43 ± 1^{c}	n.d.^{a}
**44**	(9´Z)-lycopene isomer 5	18 ± 0^{c}	19 ± 1^{c}	12 ± 2^{b}	n.d.^{a}	n.d.^{a}	n.d.^{a}	n.d.^{a}
**45**	(all-*E)*-lycopene	421 ± 1^{d}	119 ± 2^{b}	441 ± 3^{d}	887 ± 3^{f}	98 ± 2^{a}	457 ± 0^{e}	351 ± 1^{c}
**46**	(Z)-lycopene isomer 6	37 ± 2^{b}	n.d.^{a}	n.d.^{a}	n.d.^{a}	n.d.^{a}	100 ± 4^{c}	n.d.^{a}
**Total free xanthophylls**		121 ± 6^{a}	939 ± 9^{g}	357 ± 1^{d}	211 ± 3^{c}	415 ± 7^{e}	554 ± 5^{f}	153 ± 4^{b}
**Total hydrocarbon carotenoids**		943 ± 31^{c}	618 ± 1^{b}	1117 ± 1^{d}	1246 ± 2^{e}	654 ± 3^{b}	1092 ± 18^{c}	553 ± 2^{a}
**Total xanthophyll esters**		1085 ± 27^{f}	160 ± 3^{a}	788 ± 1^{d}	739 ± 6^{c}	887 ± 21^{e}	735 ± 8^{c}	462 ± 2^{b}
**Total carotenoids**		2148 ± 64^{d}	1717 ± 11^{b}	2263 ± 2^{e}	2196 ± 7^{d}	1956 ± 31^{c}	2381 ± 31^{f}	1169 ± 7^{a}
**RAE**		23 ± 1^{b}	22 ± 0^{b}	35 ± 0^{d}	33 ± 0^{c}	48 ± 0^{e}	35 ± 0^{d}	16 ± 0^{a}

n.d. not detected (detection limit: 0.08 μg/g). Numbers correspond with the HPLC-DAD chromatogram peaks (Figure 1 and Figure S1). Results are expressed as the mean ± standard deviation of duplicate analysis (n = 2) of samples from freeze-dried papaya HHP treated pulp. Different superscript letters indicate statistically significant differences of specific content of each compound evaluated ($p \leqslant 0.05$), between treatments and the control (untreated) sample. Retinol activity equivalents are calculated according to guidelines of the United States (US) Institute of Medicine [41].
