# Supplementary material for: Effect of High Hydrostatic Pressure on the Extractability and Bioaccessibility of Carotenoids and Their Esters from Papaya (Carica papaya L.) and Its Impact on Tissue Microstructure
Source: Foods. 2021 Oct 13;10(10):2435. doi: 10.3390/foods10102435 (PMC8535580; doi:10.3390/foods10102435)
Supplement: Supplementary file 1 [file foods-10-02435-s001.zip › Supplementary Table S5 (2).pdf]

Table S5. Carotenoid content[1] (µg/100 g fresh weight) ± standard deviation of papaya (*Carica papaya* L.) pulp cv. Sweet Mary submitted to HHP treatments (100, 350, 600 MPa at CUT and 5 min) after each phase of *in vitro* simulated gastrointestinal digestion.

Compound	Phase (digesta)	Non-treated	100 MPa/ CUT	100 MPa/ 5 min	350 MPa/ CUT	350 MPa/ 5 min	600 MPa/ CUT	600 MPa/ 5 min
**Free xanthophylls**								
	Oral	6.2 ± 0.1 ^{Aa}	17.6 ± 0.4 ^{Ac}	17.5 ± 0.3 ^{Ac}	11.8 ± 0.1 ^{Ab}	16.2 ± 0.4 ^{Ac}	24.9 ± 1.8 ^{Ad}	23.4 ± 0.6 ^{Ad}
**(all-*E*)-violaxanthin**	Gastric	40.0 ± 2.3 ^{Ba}	20.8 ± 0.1 ^{Ba}	39.8 ± 0.1 ^{Ba}	21.1 ± 0.5 ^{Ba}	75.1 ± 0.6 ^{Ca}	28.6 ± 1.9 ^{Aa}	46.1 ± 0.5 ^{Ca}
	Intestinal	34.3 ± 0.7 ^{Ba}	31.6 ± 0.1 ^{Ca}	30.6 ± 4.1 ^{ABa}	33.8 ± 0.2 ^{Ca}	37.6 ± 1.1 ^{Ba}	35.1 ± 2.2 ^{Aa}	30.7 ± 1.4 ^{Ba}
	Oral	4.6 ± 0.2 ^{Aa}	4.5 ± 0.3 ^{Aa}	9.7 ± 0.4 ^{Bc}	11.3 ± 0.3 ^{Cd}	5.0 ± 0.3 ^{Aa}	7.8 ± 0.6 ^{Ab}	13.3 ± 0.2 ^{Be}
**(all-*E*)-zeaxanthin**	Gastric	18.8 ± 0.8 ^{Cd}	9.6 ± 0.8 ^{Bc}	10.6 ± 0.4 ^{Bc}	7.6 ± 0.2 ^{Bb}	5.9 ± 0.2 ^{Ab}	2.91 ± 0.1 ^{Aa}	10.9 ± 0.4 ^{Ac}
	Intestinal	13.7 ± 0.4 ^{Bd}	8.0 ± 0.7 ^{Bb}	5.4 ± 0.3 ^{Aa}	5.0 ± 0.0 ^{Aa}	10.9 ± 0.1 ^{Bc}	13.6 ± 1.3 ^{Ad}	13.1 ± 0.2 ^{Bd}
	Oral	26.9 ± 0.3 ^{Cd}	7.0 ± 0.5 ^{Ab}	tr. ^{Aa}	tr. ^{Aa}	tr. ^{Aa}	tr. ^{Aa}	9.6 ± 0.1 ^{Cc}
**(all-*E*)-antheraxanthin**	Gastric	9.6 ± 0.3 ^{Ac}	7.5 ± 0.5 ^{Ab}	tr. ^{Aa}	tr. ^{Aa}	tr. ^{Aa}	tr. ^{Aa}	8.2 ± 0.3 ^{Bb}
	Intestinal	16.5 ± 0.7 ^{Bd}	13.3 ± 0.7 ^{Bc}	tr. ^{Aa}	tr. ^{Aa}	tr. ^{Aa}	tr. ^{Aa}	5.2 ± 0.1 ^{Ab}
	Oral	201.1 ± 0.3 ^{Bc}	9.8 ± 0.0 ^{Ba}	9.2 ± 0.2 ^{Ba}	30.6 ± 0.2 ^{Cd}	20.3 ± 0.1 ^{Bb}	43.3 ± 0.3 ^{Ce}	10.0 ± 0.6 ^{Ba}
**(all-*E*)-β-cryptoxanthin**	Gastric	19.8 ± 0.5 ^{Ad}	4.6 ± 0.0 ^{Aab}	3.7 ± 0.1 ^{Aa}	18.3 ± 0.4 ^{Ac}	18.8 ± 0.1 ^{Acd}	35.1 ± 0.1 ^{Be}	5.0 ± 0.3 ^{Ab}
	Intestinal	25.5 ± 0.1 ^{Cc}	30.6 ± 0.0 ^{Ce}	53.4 ± 0.3 ^{Cf}	24.0 ± 0.2 ^{Bb}	27.4 ± 0.2 ^{Cd}	31.1 ± 0.6 ^{Ae}	11.3 ± 0.1 ^{Ba}
**Total free xanthophylls[2]**								
**Oral phase**		60 ± 1^{Ac}	39 ± 1^{Aa}	36 ± 3^{Aa}	54 ± 1^{Bb}	42 ± 1^{Aa}	76 ± 2^{ABd}	56 ± 2^{Abc}
**Gastric phase**		88 ± 6^{Bd}	42 ± 1^{Ba}	54 ± 5^{Bb}	47 ± 1^{Aab}	100 ± 1^{Ce}	67 ± 2^{Ac}	70 ± 1^{Bc}
**Intestinal phase**		90 ± 1^{Bc}	84 ± 0^{Cbc}	89 ± 5^{Cc}	63 ± 1^{Ca}	76 ± 2^{Bb}	80 ± 4^{Bb}	60 ± 2^{Aa}
**Xanthophyll esters**								
	Oral	26.4 ± 1.3 ^{Ac}	212.7 ± 0.8 ^{Cd}	tr. ^{Aa}	tr. ^{Aa}	12.9 ± 0.7 ^{Bb}	tr. ^{Aa}	269.0 ± 2.6 ^{Ce}
**(all-*E*)-lutein-3-O-myristate**	Gastric	36.6 ± 1.4 ^{Bc}	100.5 ± 0.5 ^{Bd}	tr. ^{Aa}	tr. ^{Aa}	7.7 ± 0.2 ^{Ab}	tr. ^{Aa}	106.8 ± 0.0 ^{Ae}
	Intestinal	46.1 ± 0.2 ^{Cc}	25.3 ± 0.7 ^{Ab}	tr. ^{Aa}	tr. ^{Aa}	150.1 ± 0.5 ^{Ce}	tr. ^{Aa}	145.5 ± 0.0 ^{Bd}
	Oral	20.8 ± 0.2 ^{Ab}	62.2 ± 1.5 ^{Ce}	tr. ^{Aa}	50.8 ± 0.2 ^{Cd}	30.7 ± 1.3 ^{Ac}	53.1 ± 0.2 ^{Cd}	51.2 ± 0.1 ^{Cd}
**(9Z)-violaxanthin dimyristate**	Gastric	46.5 ± 1.1 ^{Cd}	39.4 ± 0.2 ^{Bc}	tr. ^{Aa}	33.2 ± 0.1 ^{Ab}	74.5 ± 2.8 ^{Be}	36.7 ± 0.1 ^{Abc}	40.5 ± 0.3 ^{Bc}
	Intestinal	26.73 ± 0.1 ^{Bc}	tr. ^{Aa}	tr. ^{Aa}	39.9 ± 0.6 ^{Be}	32.4 ± 1.0 ^{Ad}	48.9 ± 0.3 ^{Bf}	19.0 ± 0.2 ^{Ab}
	Oral	29.0 ± 0.3 ^{Bc}	30.1 ± 0.5 ^{Cc}	tr. ^{Aa}	61.7 ± 0.4 ^{Cd}	65.1 ± 0.2 ^{Ce}	117.1 ± 1.5 ^{Cf}	15.8 ± 0.1 ^{Bb}
**(all-*E*)-antheraxanthin myristate palmitate**	Gastric	34.2 ± 1.0 ^{Cc}	20.5 ± 0.3 ^{Bb}	tr. ^{Aa}	46.2 ± 2.2 ^{Be}	46.8 ± 0.9 ^{Be}	52.0 ± 0.8 ^{Bf}	40.4 ± 0.6 ^{Cd}
	Intestinal	26.5 ± 0.5 ^{Ad}	tr. ^{Aa}	tr. ^{Aa}	24.0 ± 1.2 ^{Ac}	30.8 ± 0.6 $^{A e}$	25.1 ± 0.8 ^{Acd}	10.4 ± 0.4 ^{Ab}

**(all-*E*)-β-cryptoxanthin caprate**	Oral	37.3 ± 0.1 ^{Aa}	46.2 ± 1.2 ^{Cb}	37.7 ± 0.2 ^{Ba}	92.4 ± 0.1 ^{Cd}	114.1 ± 0.3 ^{Ce}	132.3 ± 0.8 ^{Cf}	51.9 ± 0.3 ^{Cc}
	Gastric	72.1 ± 0.2 ^{Ce}	39.9 ± 0.4 ^{Bb}	38.3 ± 0.4 ^{Ca}	65.9 ± 0.7 ^{Bd}	91.1 ± 0.2 ^{Bf}	108.2 ± 0.1 ^{Bg}	45.9 ± 0.8 ^{Bc}
	Intestinal	47.9 ± 0.8 ^{Be}	17.9 ± 0.1 ^{Ab}	tr. ^{Aa}	47.0 ± 0.4 ^{Ae}	44.7 ± 1.3 ^{Ad}	44.3 ± 0.2 ^{Ad}	39.4 ± 0.2 ^{Ac}
**(all-*E*)-lutein dimyristate**	Oral	26.1 ± 0.4 ^{Ca}	271.6 ± 1.4 ^{Cd}	14.9 ± 0.0 ^{Aa}	43.9 ± 1.6 ^{Ab}	67.1 ± 1.2 ^{Bc}	75.2 ± 1.2 ^{Ac}	264.4 ± 13.1 ^{Bd}
	Gastric	23.3 ± 0.9 ^{Ba}	162.2 ± 3.1 ^{Bf}	148.1 ± 1.0 ^{Ce}	32.9 ± 0.2 ^{Aab}	41.1 ± 0.2 ^{Ab}	101.1 ± 0.1 ^{Bc}	125.3 ± 5.5 ^{Ad}
	Intestinal	15.2 ± 0.6 ^{Aa}	80.4 ± 2.5 ^{Ac}	27.4 ± 0.1 ^{Bb}	143.5 ± 5.2 ^{Be}	216.1 ± 2.1 ^{Cf}	120.6 ± 2.0 ^{Cd}	138.4 ± 3.6 ^{Ae}
**(all-*E*)-β-cryptoxanthin laurate**	Oral	54.6 ± 0.4 ^{Aa}	128.7 ± 1.4 ^{Cc}	83.1 ± 0.2 ^{Ab}	189.4 ± 0.9 ^{Cd}	238.7± 0.4 ^{Ce}	280.2 ± 1.4 ^{Cf}	126.6 ± 4.0 ^{Bc}
	Gastric	140.6 ± 7.4 ^{Cb}	90.9 ± 0.5 ^{Ba}	81.2 ± 0.0 ^{Aa}	141.2 ± 1.0 ^{Bb}	149.0 ± 1.1 ^{Bb}	208.2 ± 0.0 ^{Bc}	88.5 ± 2.8 ^{Aa}
	Intestinal	105.0 ± 1.0 ^{Bc}	46.2 ± 0.6 ^{Aa}	166.3 ± 0.8 ^{Bf}	124.0 ± 1.3 ^{Ae}	117.0 ± 0.7 ^{Ad}	109.3 ± 1.8 ^{Ac}	97.1 ± 2.1 ^{Ab}
**all-*E*)-β-cryptoxanthin myristate**	Oral	5.2 ± 0.0 ^{Aa}	16.5 ± 0.4 ^{Cc}	10.2 ± 0.0 ^{Ab}	23.0 ± 0.2 ^{Bd}	28.9 ± 3.0 ^{Be}	32.2 ± 0.8 ^{Ce}	19.2 ± 0.2 ^{Ccd}
	Gastric	18.7 ± 1.4 ^{Ccd}	14.1 ± 0.0 ^{Bb}	30.3 ± 0.2 ^{Ce}	33.4 ± 0.2 ^{Cf}	20.5 ± 1.2 ^{Ad}	17.2 ± 0.1 ^{Bc}	8.9 ± 0.1 ^{Aa}
	Intestinal	13.6 ± 0.3 ^{Bc}	3.3 ± 0.1 ^{Aa}	22.3 ± 0.2 ^{Be}	18.5 ± 0.2 ^{Ad}	17.2 ± 0.0 ^{Ad}	8.7 ± 0.0 ^{Ab}	13.6 ± 0.4 ^{Bc}
**(all-*E*)-β-cryptoxanthin palmitate**	Oral	7.5 ± 0.0 ^{Cc}	31.9 ± 0.2 ^{Cf}	2.9 ± 0.0 ^{Bb}	9.4 ± 0.1 ^{Cd}	11.8 ± 0.1 ^{Ce}	11.6 ± 0.5 ^{Ce}	tr. ^{Aa}
	Gastric	6.8 ± 0.1 ^{Bc}	20.6 ± 0.1 ^{Be}	6.7 ± 0.1 ^{Cc}	2.1 ± 0.1 ^{Bb}	8.4 ± 0.0 ^{Bd}	7.0 ± 0.4 ^{Bc}	tr. ^{Aa}
	Intestinal	5.7 ± 0.2 ^{Ae}	4.1 ± 0.1 ^{Ac}	tr. ^{Aa}	1.2 ± 0.0 ^{Ab}	tr. ^{Aa}	4.8 ± 0.3 ^{Ad}	tr. ^{Aa}
**Total xanthophyll esters**[2]								
**Oral phase**		207 ± 3^{Ab}	800 ± 7^{Cf}	149 ± 1^{Aa}	471 ± 3^{Bc}	569 ± 7^{Bd}	702 ± 6^{Be}	798 ± 20^{Bf}
**Gastric phase**		378 ± 15^{Cb}	488 ± 5^{Bd}	375 ± 3^{Cab}	355 ± 4^{Aa}	436 ± 8^{Ac}	530 ± 2^{Ae}	456 ± 10^{Ac}
**Intestinal phase**		287 ± 4^{Bc}	177 ± 4^{Aa}	216 ± 1^{Bb}	559 ± 10^{Cf}	608 ± 8^{Cg}	528 ± 8^{Ae}	463 ± 11^{Ad}
**Hydrocarbon carotenoids**								
**(all-*E*)-α-carotene**	Oral	20.8 ± 0.4 ^{Bd}	12.9 ± 0.4 ^{Ab}	16.6 ± 1.0 ^{Bc}	9.1 ± 0.8 ^{Aa}	31.9 ± 0.9 ^{Ce}	52.1 ± 1.9 ^{Cf}	13.7 ± 0.1 ^{Cbc}
	Gastric	23.4 ± 0.8 ^{Cc}	25.4 ± 0.2 ^{Bc}	8.5 ± 0.2 ^{Aa}	15.3 ± 1.5 ^{Bb}	23.5 ± 0.1 ^{Bc}	26.0 ± 1.9 ^{Bc}	12.1 ± 0.2 ^{Bb}
	Intestinal	6.6 ± 0.2 ^{Aa}	63.6 ± 0.8 ^{Cd}	76.5 ± 0.5 ^{Ce}	12.0 ± 1.3 ^{ABbc}	9.4 ± 0.3 ^{Aab}	15.4 ± 0.6 ^{Ac}	7.6 ± 0.1 ^{Aab}
**(all-*E*)-β-carotene**	Oral	77.6 ± 0.4 ^{Aa}	101.3 ± 0.9 ^{Cb}	82.2 ± 1.4 ^{Ba}	197.5 ± 1.7 ^{Cc}	304.2 ± 12.2 ^{Cd}	350.1 ± 2.7 ^{Ce}	105.2 ± 2.1 ^{Cb}
	Gastric	107.6 ± 1.5 ^{Bd}	57.0 ± 0.2 ^{Bb}	49.3 ± 0.0 ^{Aa}	123.2 ± 0.2 ^{Be}	112.2 ± 4.2 ^{Ad}	177.8 ± 0.3 ^{Bf}	74.9 ± 1.4 ^{Ac}
	Intestinal	118.1 ± 0.6 ^{Ce}	12.6 ± 0.2 ^{Aa}	107.1 ± 2.1 ^{Ccd}	110.6 ± 1.0 ^{Ad}	147.0 ± 3.0 ^{Bf}	101.2 ± 0.9 ^{Ac}	89.3 ± 1.7 ^{Bb}
**(13Z)-lycopene isomer 2**	Oral	46.3 ± 0.3 ^{Aa}	214.9 ± 0.9 ^{Bg}	95.8 ± 1.8 ^{Ac}	67.2 ± 0.8 ^{Ab}	134.1 ± 0.8 ^{Ae}	191.5 ± 1.6 ^{Cf}	129.8 ± 2.2 ^{Cd}
	Gastric	77.8 ± 3.3 ^{Ca}	214.1 ± 3.2 ^{Be}	188.4 ± 2.9 ^{Cd}	77.1 ± 0.7 ^{Ba}	197.5 ± 1.0 ^{Bd}	156.8 ± 0.4 ^{Bc}	112.8 ± 0.5 ^{Bb}
	Intestinal	69.5 ± 1.1 ^{Ba}	76.2 ± 1.1 ^{Aa}	120.2 ± 4.4 ^{Bb}	186.6 ± 1.2 ^{Cc}	230.9 ± 3.3 ^{Cd}	127.1 ± 2.9 ^{Ab}	73.7 ± 1.4 ^{Aa}
**(9Z)-lycopene isomer 4**	Oral	8.6 ± 0.2 ^{Aa}	19.3 ± 0.2 ^{Bc}	44.9 ± 1.4 ^{Bd}	20.6 ± 0.4 ^{Ac}	15.7 ± 0.2 ^{Ab}	9.7 ± 0.3 ^{Aa}	9.5 ± 0.2 ^{Aa}
	Gastric	9.2 ± 1.0 ^{Aa}	31.8 ± 0.0 ^{Cd}	58.1 ± 2.1 ^{Cf}	23.1 ± 0.6 ^{Ac}	39.0 ± 1.2 ^{Ce}	16.2 ± 0.1 ^{Bb}	13.4 ± 0.9 ^{Ab}

	Intestinal	8.9 ± 0.3 ^{Aa}	11.3 ± 0.2 ^{Aa}	10.6 ± 0.1 $^{Aa;}$	28.0 ± 0.9 ^{Be}	25.8 ± 0.6 ^{Bd}	23.2 ± 0.5 ^{Cc}	20.1 ± 1.1 ^{Bb}
	Oral	21.1 ± 0.4 ^{Aa}	1147.6 ± 10.8 ^{Ab}	1215.6 ± 7.3 ^{Ac}	1271.2 ± 20.9 ^{Cd}	2201.4 ± 8.6 ^{Be}	2227.8 ± 9.8 ^{Ce}	2605.5 ± 12.5 ^{Cf}
**(all-*E*)-lycopene**	Gastric	436.3 ± 3.8 ^{Ba}	1338.4 ± 8.9 ^{Bf}	1490.9 ± 5.2 ^{Bg}	1180.8 ± 3.8 ^{Be}	627.5 ± 4.4 ^{Ab}	1029.3 ± 0.9 ^{Bd}	707.3 ± 8.0 ^{Ac}
	Intestinal	476.9 ± 2.8 ^{Cb}	1352.9 ± 11.5 ^{Bd}	2860.0 ± 7.7 ^{Cg}	929.5 ± 2.3 ^{Ac}	2225.0 ± 4.9 ^{Bf}	279.7 ± 5.1 ^{Aa}	1898.6 ± 4.7 ^{Be}
	Oral	29.5 ± 0.2 ^{Cc}	310.9 ± 4.4 ^{Cg}	116.8 ± 1.8 ^{Be}	124.8 ± 0.8 ^{Cf}	7.8 ± 0.4 ^{Ab}	tr. ^{Aa}	51.6 ± 0.6 ^{Bd}
**(Z)-lycopene isomer 6**	Gastric	18.3 ± 0.3 ^{Bb}	191.1 ± 0.1 ^{Be}	189.4 ± 2.7 ^{Ce}	39.0 ± 0.6 ^{Ac}	13.9 ± 0.5 ^{Bb}	tr. ^{Aa}	80.3 ± 0.2 ^{Cd}
	Intestinal	8.1 ± 0.1 ^{Ab}	126.9 ± 0.6 ^{Af}	75.0 ± 0.4 ^{Ae}	71.1 ± 1.8 ^{Bd}	23.9 ± 1.2 ^{Cc}	tr. ^{Aa}	tr. ^{Aa}
**Total hydrocarbon carotenoids**[2]								
**Oral phase**		204 ± 2^{Aa}	1807 ± 18^{Bd}	1572 ± 15^{Ab}	1690 ± 25^{Cc}	2695 ± 23^{Ce}	2831 ± 16^{Cf}	2915 ± 19^{Cg}
**Gastric phase**		673 ± 10^{Ba}	1858 ± 13^{Ce}	1985 ± 13^{Bf}	1458 ± 6^{Bd}	1014 ± 12^{Ab}	1406 ± 4^{Bc}	1001 ± 11^{Ab}
**Intestinal phase**		688 ± 5^{Bb}	1644 ± 14^{Ad}	3249 ± 18^{Cg}	1338 ± 7^{Ac}	2662 ± 13^{Bf}	547 ± 10^{Aa}	2089 ± 10^{Be}
**Total carotenoids**[3]								
**Oral phase**		471 ± 6^{Aa}	2646 ± 26^{Cd}	1757 ± 18^{Ab}	2215 ± 29^{Cc}	3306 ± 31^{Be}	3609 ± 25^{Cf}	3770 ± 41^{Cg}
**Gastric phase**		1139 ± 31^{Ca}	2388 ± 18^{Be}	2413 ± 22^{Be}	1860 ± 11^{Ac}	1550 ± 21^{Ab}	2003 ± 7^{Bd}	1527 ± 23^{Ab}
**Intestinal phase**		1065 ± 10^{Ba}	1904 ± 19^{Ac}	3555 ± 24^{Cg}	1959 ± 19^{Bd}	3346 ± 24^{Cf}	1154 ± 22^{Ab}	2613 ± 22^{Be}

tr.: traces.

[1] Results are expressed as the mean ± standard deviation (n = 4). This came from obtaining at least two independent extracts (n = 2) and performing HPLC determinations of each treatment (n = 2). Superscript capital letters indicate statistically significant differences ($p \leq 0.05$) between digestion phases. Superscript small letters indicate statistically significant differences ($p \leq 0.05$) between treatment.

[2] Represents the algebraic sum of the most representative free xanthophylls, xanthopyhll esters and hydrocarbon carotenoids, respectivelly.

[3] Represents the algebraic sum of the most representative carotenoids identified in each sample.
