# Supplementary material for: Effect of High Hydrostatic Pressure on the Extractability and Bioaccessibility of Carotenoids and Their Esters from Papaya (Carica papaya L.) and Its Impact on Tissue Microstructure
Source: Foods. 2021 Oct 13;10(10):2435. doi: 10.3390/foods10102435 (PMC8535580; doi:10.3390/foods10102435)
Supplement: Supplementary file 1 [file foods-10-02435-s001.zip › Supplementary Tables and Figures legends (3).pdf]

Supplementary Tables Legends

Supplementary Table S1. Physical-chemical characteristics in Sweet Mary, Alicia and Eksotika papaya (*Carica papaya* L.) varieties. [1]Values are the mean of three independent determinations ± standard deviation. Superscript letter indicates statistically significant differences ($p \leq 0.05$). [2] g citric acid/100 g fresh weight.

Supplementary Table S2. Average of HHP extraction parameters[1] applied to extract carotenoids from papaya (*Carica papaya* L.) Sweet Mary, Alicia and Eksotika varieties from the Canary Islands (Spain). [1] Target hold pressure and time-extraction pressure and time that was anticipated at hold time and set by command console of the high pressure unit. Target extraction temperature-anticipated temperature of entire extraction during entire length of extraction time for one batch of samples. AUC pressure-the size of area under the pressure curve for one batch of samples during entire length of extraction. AUC temperature the size of area under the temperature curve for one batch of samples during entire length of extraction. Δt-entire length of extraction time for one batch of samples. Mean effective influence of pressure Pextr. - influence of pressure for entire extraction obtain by dividing the AUC pressure by Δt. Mean effective influence of temperature Textr. - influence of temperature for entire extraction obtain by dividing the AUC temperature by Δt. Mean pressure at hold time – average value of pressure measured by probe within HPE cylinder during the hold time. Mean temperature at hold time – average value of temperature measured by probe within HPE cylinder during the hold time. Δt at hold time – length of hold time during which oscillation in target extraction pressure were less than 5% from what was inputted in the command console. The above parameters were calculated following the indications reported by Putnik et al. [61].

Supplementary Table S3. Carotenoid content (μg/100 g fresh weight) ± standard deviation and retinol activity equivalents (RAE) of direct pulp extracts of papaya (*Carica papaya* L.) Alicia variety submitted to HHP. n.d. not detected (detection limit: 0.08 μg/g). Numbers

correspond with the HPLC-DAD chromatogram peaks (Figure 1 and Figure S1). Results are expressed as the mean ± standard deviation of duplicate analysis (n = 2) of samples from freeze-dried papaya HHP treated pulp. Different superscript letters indicate statistically significant differences of specific content of each compound evaluated ($p \leq 0.05$), between treatments and the control (untreated) sample. Retinol activity equivalents are calculated according to guidelines of the United States (US) Institute of Medicine [41].

Supplementary Table S4. Carotenoid content (μg/100 g fresh weight) ± standard deviation and retinol activity equivalents (RAE) of direct pulp extracts of papaya (*Carica papaya* L.) Eksotika variety submitted to HHP. n.d. not detected (detection limit: 0.08 μg/g). Numbers correspond with the HPLC-DAD chromatogram peaks (Figure 1 and Figure S1). Results are expressed as the mean ± standard deviation of duplicate analysis (n = 2) of samples from freeze-dried papaya HHP treated pulp. Different superscript letters indicate statistically significant differences of specific content of each compound evaluated ($p \leq 0.05$), between treatments and the control (untreated) sample. Retinol activity equivalents are calculated according to guidelines of the United States (US) Institute of Medicine [41].

Supplementary Table S5. Carotenoid content[1] (μg/100 g fresh weight) ± standard deviation of papaya (*Carica papaya* L.) pulp cv. Sweet Mary submitted to HHP treatments (100, 350, 600 MPa at CUT and 5 min) after each phase of *in vitro* simulated gastrointestinal digestion. tr.: traces. [1]Results are expressed as the mean ± standard deviation (n = 4). This came from obtaining at least two independent extracts (n = 2) and performing HPLC determinations of each treatment (n = 2). Superscript capital letters indicate statistically significant differences ($p \leq 0.05$) between digestion phases. Superscript small letters indicate statistically significant differences ($p \leq 0.05$) between treatment. [2]Represents the algebraic sum of the most representative free xanthophylls, xanthopyhll esters and hydrocarbon carotenoids, respectivelly. [3]Represents the algebraic sum of the most representative carotenoids identified in each sample.

Supplementary Figures Legends

Supplementary Fig S1. C_{30} reversed-phase chromatograms of carotenoids at 450 nm obtained from Sweet Mary papaya (*Carica papaya* L.) variety of direct (non-saponified) and saponified extracts of (a and b) control, (c and d) 350 MPa/CUT and (e and f) 350 MPa/5 min samples. Peak identities are showed in Table 1. I.S. Internal standard.

Supplementary Fig S2. Carotenoid content (μg carotenoids/100 g fresh weight) in direct extracts of (a) cv. Sweet Mary, (b) cv. Alicia and (c) cv. Eksotika papaya (*Carica papaya* L.) pulps submitted to high hydrostatic pressure [HHP; 100, 350, 600 MPa at CUT and 5 min].

Supplementary Fig S3. Carotenoid content (μg carotenoids/100 g fresh weight) in saponified extracts of (a) cv. Sweet Mary, (b) cv. Alicia and (c) cv. Eksotika papaya (*Carica papaya* L.) pulps submitted to high hydrostatic pressure [HHP; 100, 350, 600 MPa at CUT and 5 min].

Supplementary Fig S4. C_{30} reversed-phase chromatograms of carotenoids at 450 nm obtained from cv. Sweet Mary papaya (*Carica papaya* L.) pulp after *in vitro* gastrointestinal digestion (micellar fraction) of untreated (control) sample (a) oral phase; (b) gastric phase; (c) intestinal phase and submitted to high hydrostatic pressure (350 MPa/5min) sample (d) oral phase, (e) gastric phase and (f) intestinal phase. Peak identities in Table 1.

Supplementary Fig S5. Optical microscopy of Sweet Mary papaya (*Carica papaya* L.) variety of untreated pulps and submitted to 100, 350, 600 MPa at CUT and 5 min after *in vitro* simulated gastrointestinal digestion of micellar fractions. Mi: micelle.
