# Supplementary figures and images for: Effect of High Hydrostatic Pressure on the Extractability and Bioaccessibility of Carotenoids and Their Esters from Papaya (Carica papaya L.) and Its Impact on Tissue Microstructure
Source: Foods. 2021 Oct 13;10(10):2435. doi: 10.3390/foods10102435 (PMC8535580; doi:10.3390/foods10102435)

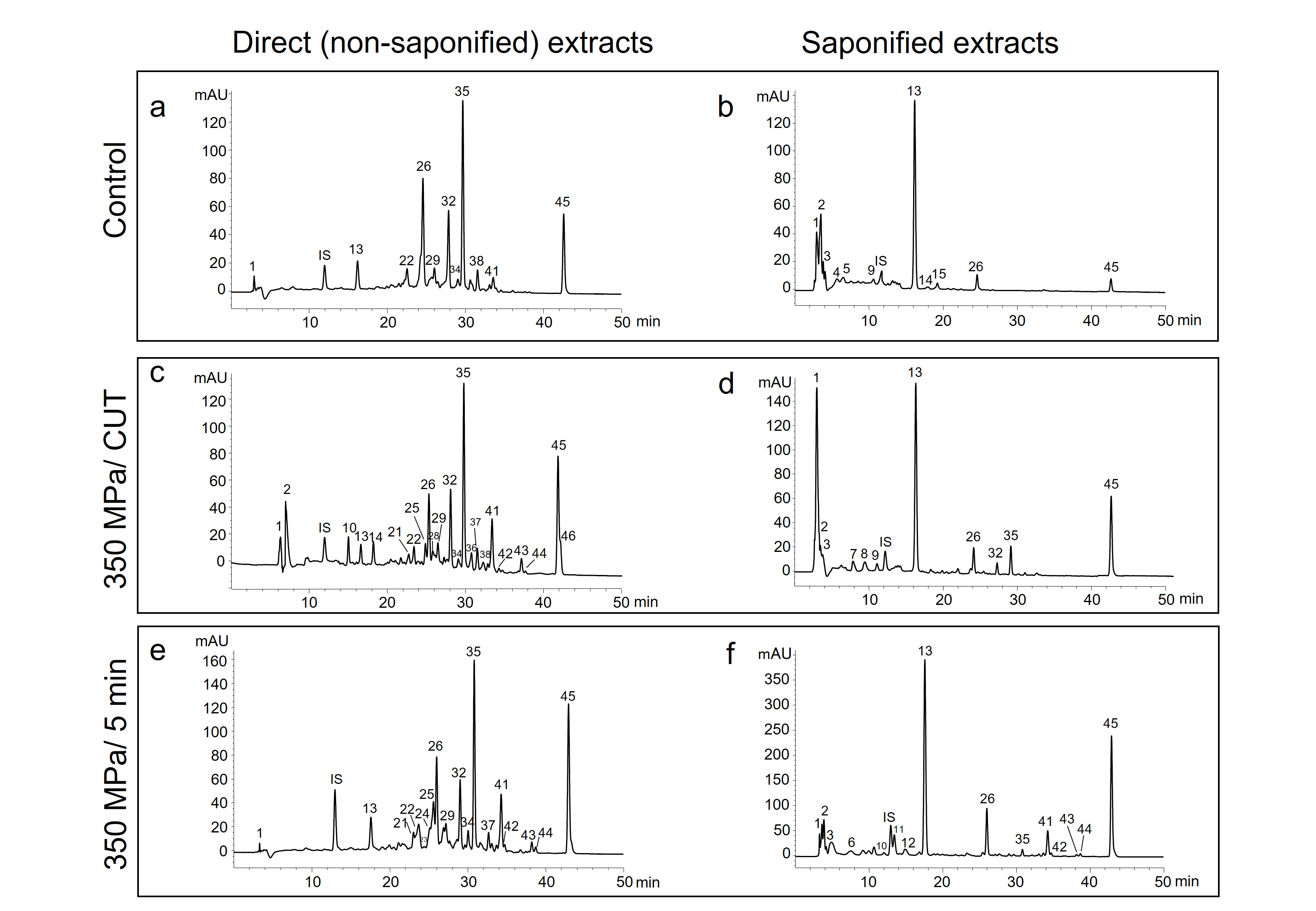

Supplement: Supplementary file 1 [file foods-10-02435-s001.zip › Supplementary Fig S1 (6).tif]

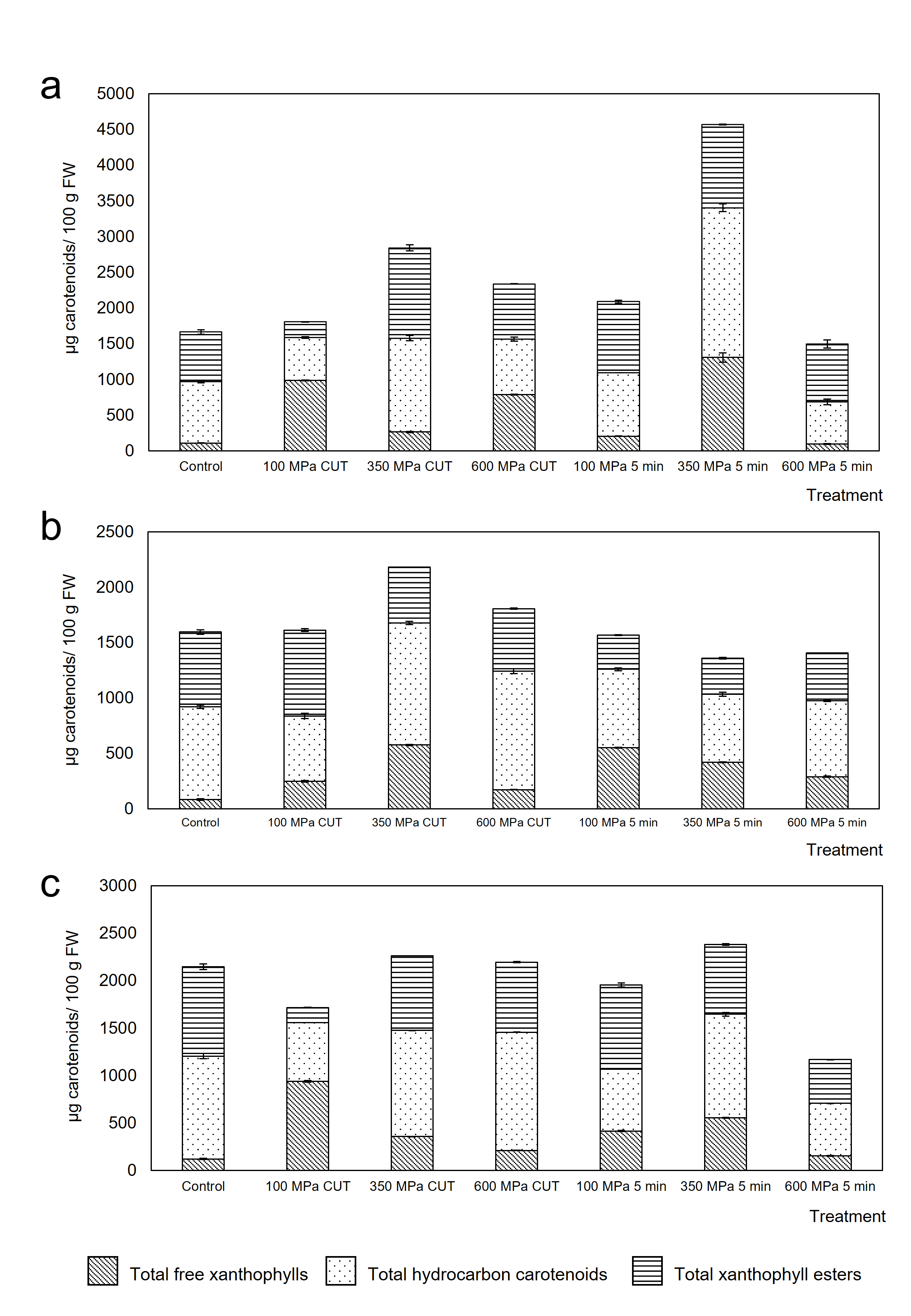

Supplement: Supplementary file 1 [file foods-10-02435-s001.zip › Supplementary Fig S2 (5).tif]

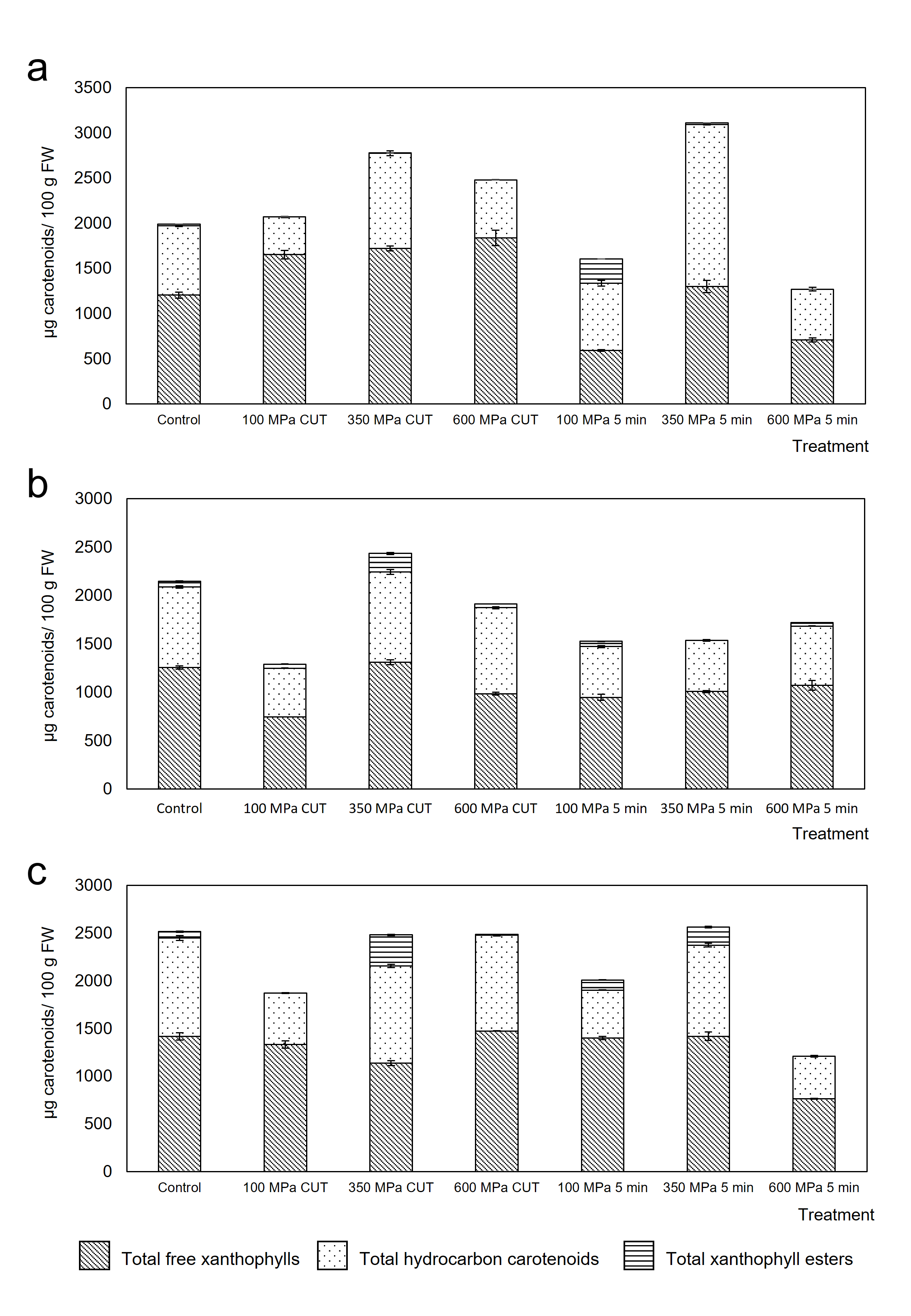

Supplement: Supplementary file 1 [file foods-10-02435-s001.zip › Supplementary Fig S3 (5).tif]

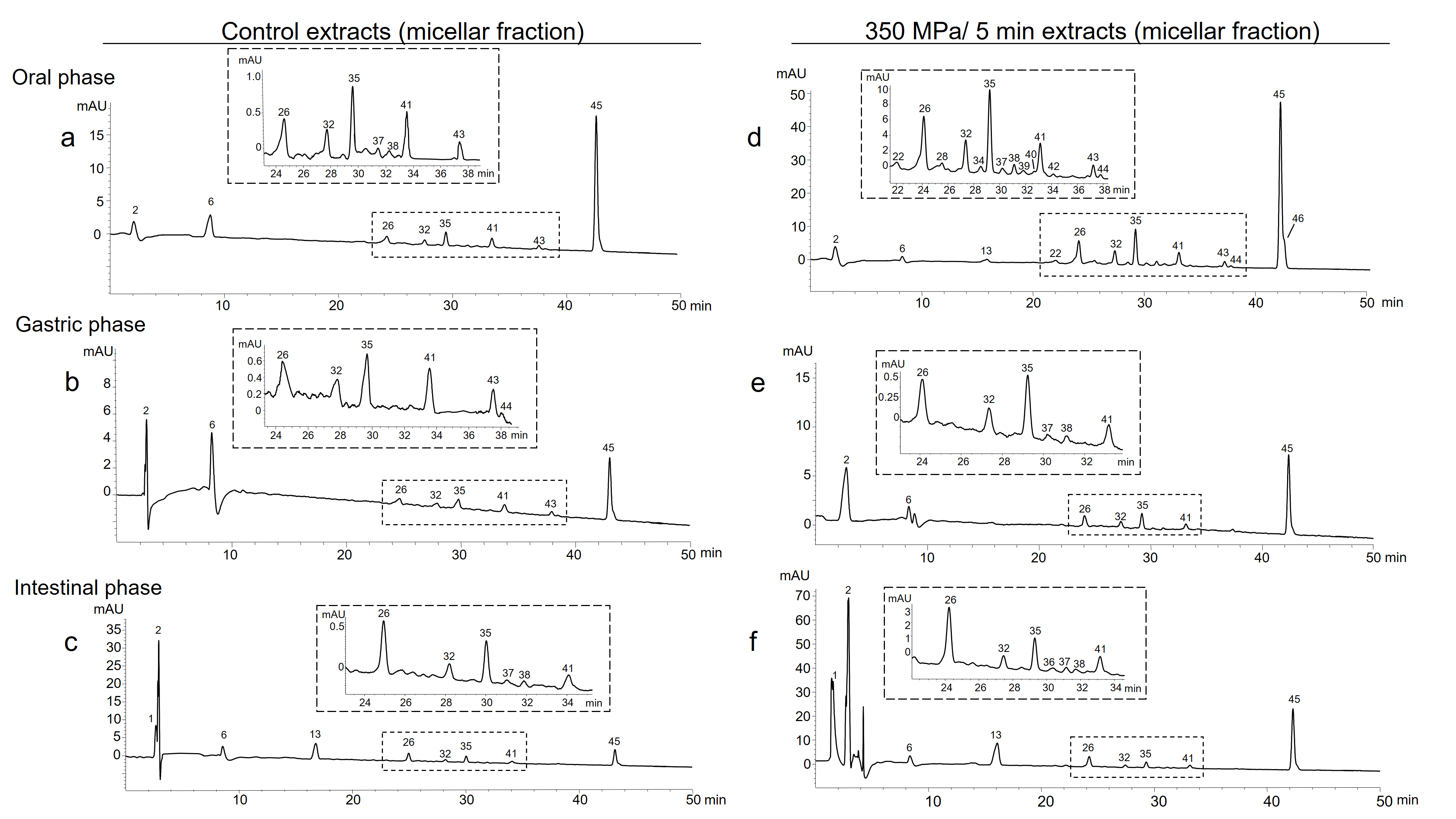

Supplement: Supplementary file 1 [file foods-10-02435-s001.zip › Supplementary Fig S4 (4).tif]

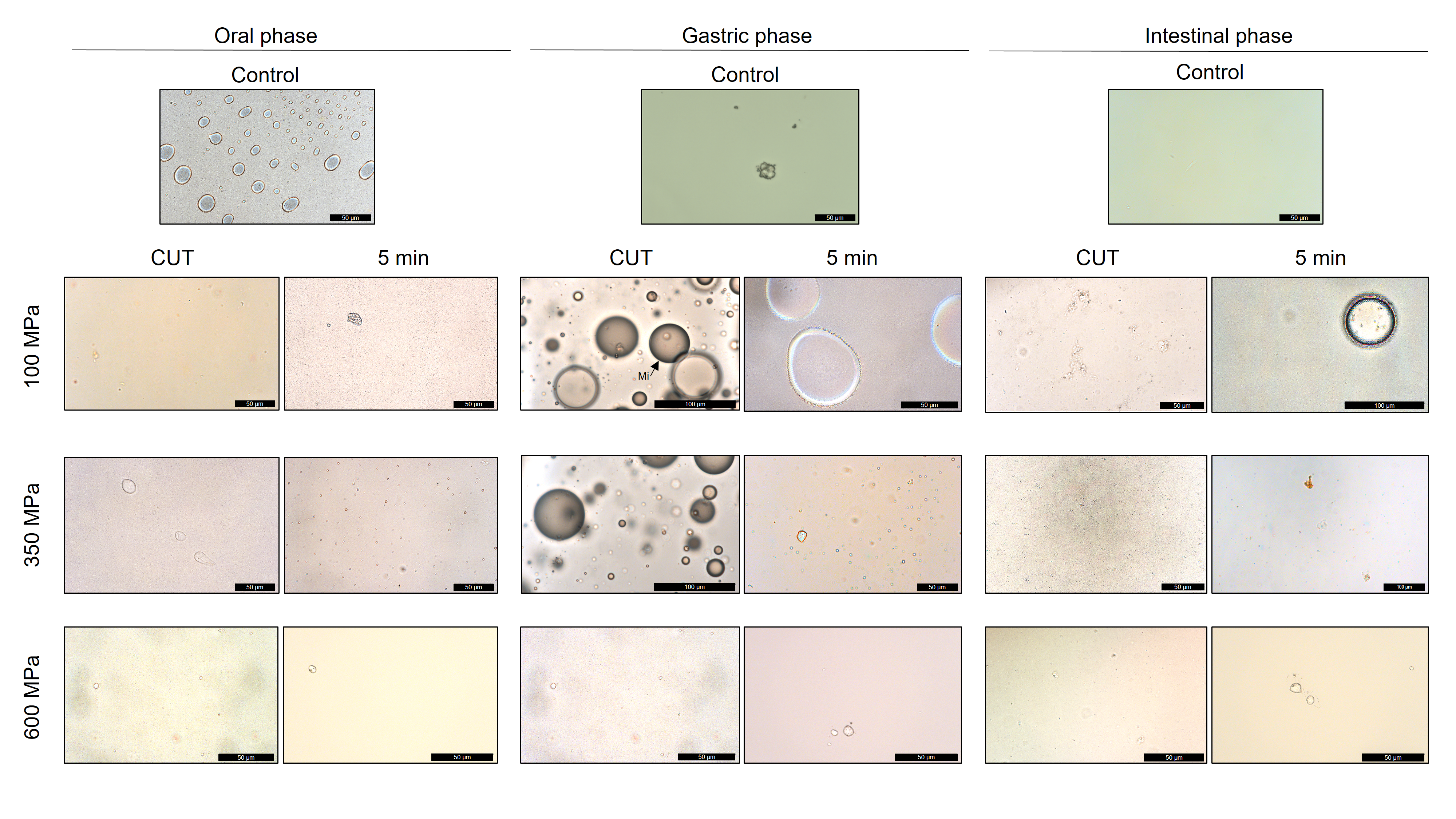

Supplement: Supplementary file 1 [file foods-10-02435-s001.zip › Supplementary Fig S5 (2).tif]
